# The Apicomplexa-specific glucosamine-6-phosphate *N*-acetyltransferase gene family encodes a key enzyme for glycoconjugate synthesis with potential as therapeutic target

**DOI:** 10.1038/s41598-018-22441-3

**Published:** 2018-03-05

**Authors:** Marta Cova, Borja López-Gutiérrez, Sara Artigas-Jerónimo, Aida González-Díaz, Giulia Bandini, Steven Maere, Lorenzo Carretero-Paulet, Luis Izquierdo

**Affiliations:** 1 ISGlobal, Barcelona Ctr. Int. Health Res. (CRESIB), Hospital Clínic – Universitat de Barcelona, Barcelona, Spain; 2 Department of Molecular and Cell Biology, Boston University Goldman School of Dental Medicine, Boston, USA; 3 Ghent University, Department of Plant Biotechnology and Bioinformatics, B-9052 Ghent, Belgium; 4 VIB Center for Plant Systems Biology, B-9052 Ghent, Belgium; 5 Bioinformatics Institute Ghent, Ghent University, B-9052 Ghent, Belgium

## Abstract

Apicomplexa form a phylum of obligate parasitic protozoa of great clinical and veterinary importance. These parasites synthesize glycoconjugates for their survival and infectivity, but the enzymatic steps required to generate the glycosylation precursors are not completely characterized. In particular, glucosamine-phosphate *N*-acetyltransferase (GNA1) activity, needed to produce the essential UDP-*N*-acetylglucosamine (UDP-GlcNAc) donor, has not been identified in any Apicomplexa. We scanned the genomes of *Plasmodium falciparum* and representatives from six additional main lineages of the phylum for proteins containing the Gcn5-related *N*-acetyltransferase (GNAT) domain. One family of GNAT-domain containing proteins, composed by a *P. falciparum* sequence and its six apicomplexan orthologs, rescued the growth of a yeast temperature-sensitive GNA1 mutant. Heterologous expression and *in vitro* assays confirmed the GNA1 enzymatic activity in all lineages. Sequence, phylogenetic and synteny analyses suggest an independent origin of the Apicomplexa-specific GNA1 family, parallel to the evolution of a different GNA1 family in other eukaryotes. The inability to disrupt an otherwise modifiable gene target suggests that the enzyme is essential for *P. falciparum* growth. The relevance of UDP-GlcNAc for parasite viability, together with the independent evolution and unique sequence features of Apicomplexa GNA1, highlights the potential of this enzyme as a selective therapeutic target against apicomplexans.

## Introduction

Apicomplexa form one of the largest and most diverse phyla of obligate intracellular parasites in the protist kingdom. They exhibit a fascinating biology, featuring (i) a specialized set of microtubules at the apical end of the cell, (ii) plastid-derived organelle called apicoplast, lost in some lineages, and (iii) a wide variety of morphologies and complex life cycles, consistent with the ability to infect almost every kind of animal from mollusks to mammals^1^. Although it has been estimated that between 1.2 and 10 million species exist^2^, only about 5,000–6,000 have been identified to date^1^, including species of clinical importance. *Plasmodium* species, for example, cause malaria, a disease responsible for more than 200 million new cases and 445,000 deaths in 2016^3^. Toxoplasmosis, caused by the ubiquitous human pathogen *Toxoplasma gondii*, contributes to congenital disease and opportunistic infections in immunocompromised persons^4^. *Cryptosporidium parvum* is responsible for cryptosporidiosis, the second leading cause of diarrheal disease in infants in developing countries^5^. Other Apicomplexa species are also very relevant to food security and veterinary medicine, since organisms like *Babesia* or *Theileria spp*. infect cattle, and *Eimeria spp*. infects poultry, producing severe economic losses every year^6,7^. Furthermore, their hosts are not restricted to vertebrates, as in *Gregarina niphandrodes*, which commonly infects beetles^8^. However, despite the global economic burden and major human health problems caused by Apicomplexa, many aspects of their biology and host interaction are still unknown. Some of the reasons that may be hindering the characterization of specific biochemical pathways in these organisms are possibly linked to their evolutionary transition from a free-living to an obligate parasitic lifestyle, including (i) the massive loss of genes involved in diverse metabolic processes^9^, and (ii) their ability to scavenge metabolite precursors from their hosts^10^.

Glycoconjugates cover the surface of many protozoan parasites, forming protective barriers and mediating host-pathogen interactions^11^. Glycosylphosphatidylinositol (GPI) anchors, attached to the C-terminus of proteins to anchor them to cell membranes, are the major glycan moieties identified on the surface of *P. falciparum* and other Apicomplexa^12,13^. GPI anchors are composed of a conserved glycan backbone linked to a *myo*-inositol ring of phosphatidylinositol (PI)^14^. GPI synthesis involves the initial transfer of *N*-acetylglucosamine (GlcNAc) from the UDP-GlcNAc donor to the PI acceptor followed by GlcNAc de-acetylation^15^. Apicomplexa also *N*-glycosylate proteins in the endoplasmic reticulum^16,17^, in a process that requires GlcNAc transfer from UDP-GlcNAc to dolichol phosphate (Dol-P) to generate the core lipid-linked oligosaccharide precursor. Both GPI biosynthesis and *N*-glycosylation are highly conserved processes critical for eukaryotic cell viability^18–21^. UDP-GlcNAc is also donor for the nucleocytosolic *O*-GlcNAc transferase, characterized in *C. parvum*^22^, and it serves as intermediate for the generation of UDP-*N*-acetylgalactosamine, required for the *O*-glycosylation of mucin-like domains in *C. parvum* and *T. gondii*^23,24^. Due to the importance of this precursor, blocking UDP-GlcNAc biosynthesis leads to growth arrest, or non-viable phenotypes, in species as diverse as *Trypanosoma brucei*, *Arabidopsis thaliana* and yeast^25–28^. This suggests that the UDP-GlcNAc metabolic route is essential and, therefore, a potential target for selective inhibitors.

**Figure 1:**
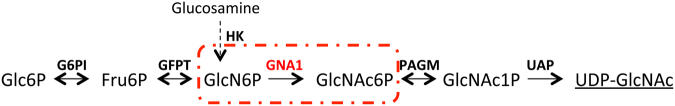
Biosynthesis of UDP-GlcNAc in Apicomplexa. Fru6P, Fructose-6-phosphate; Glc6P, Glucose-6-phosphate; G6PI, Glucose-6-phosphate isomerase (Enzyme Commission (EC) 5.3.1.9); GFPT, Glucosamine-fructose-6-phosphate aminotransferase (EC 2.6.1.16); GNA1, Glucosamine-phosphate-*N*-acetyltransferase (EC 2.3.1.4); HK, Hexokinase (EC 2.7.1.1); PAGM, Phosphoacetylglucosamine mutase (EC 5.4.2.3); UAP, UDP-*N*-acetylglucosamine pyrophosphorylase (EC 2.7.7.23). The reaction catalyzed by the GNA1 enzyme is marked with a red box.

UDP-GlcNAc biosynthesis requires the acetylation of the GlcN-6P precursor to generate GlcNAc-6P (Fig. 1)^29^. This enzymatic step is catalyzed by a glucosamine-phosphate-*N*-acetyltransferase (GNA1) activity. GNA1 belongs to the large superfamily of Gcn5-*N*-acetyltransferase domain (GNAT)-containing proteins, grouping different families of enzymes that use acetyl coenzyme A (AcCoA) to transfer an acetyl group to a substrate^30,31^. Although highly divergent at the sequence level, GNAT proteins are well-conserved in structure and catalytic mechanism^26,32,33^ and GNA1 enzymes have been identified and characterized throughout the eukaryote kingdom^26,27,33^. However, the identification of GNA1 in the genome of *P. falciparum*, or any other apicomplexan, has remained elusive^29^ despite the fact that the presence of UDP-GlcNAc was verified in different parasite stages^34–36^.

Here we identify GNA1 in *P. falciparum* and six other species representing main lineages of the phylum Apicomplexa. GNA1 forms a specific gene family with an evolutionary origin at least as ancient as the phylum. The independent evolution and unique sequence features of this enzyme, which we show is likely essential for parasite growth, highlight GNA1 as a potential pan-apicomplexan drug target amenable to selective inhibition.

## Results

### Classification of the superfamily of GNAT-domain containing proteins in Apicomplexa

**Figure 2:**
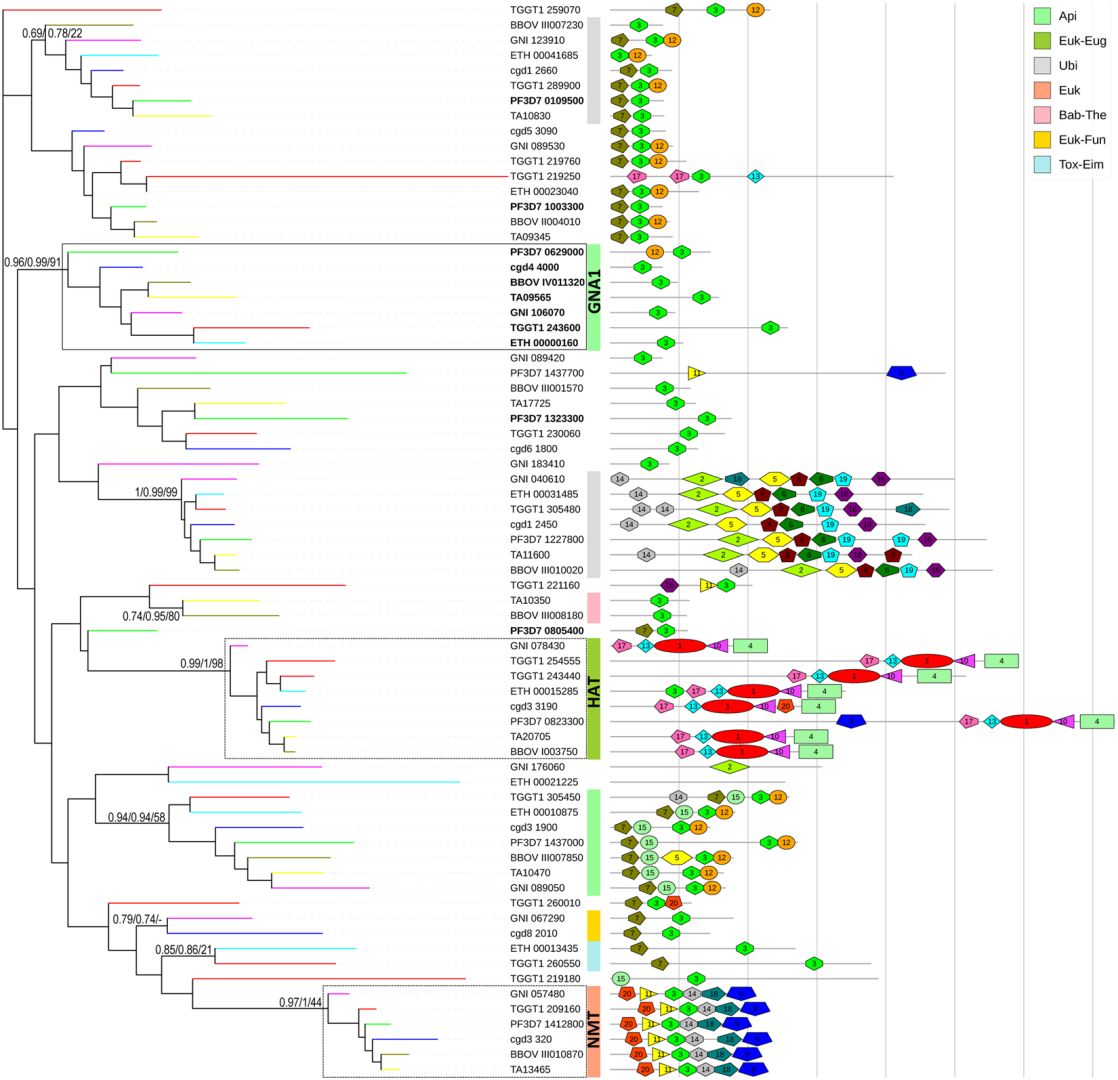
Phylogeny, protein domain architecture and taxonomic distribution of 71 GNAT proteins from seven apicomplexan species. The maximum-likelihood unrooted phylogenetic tree is drawn to scale, with branch lengths proportional to evolutionary distances between nodes. Branches leading to extant nodes in the tree are colored according to the species. Indicated in bold are sequences examined for GNA1 activity. Values next to the orthogroup-defining nodes indicate statistical support from maximum-likelihood, Bayesian and neighbor-joining phylogenetic analysis, respectively. For EupathDB orthogroups retrieved as well-supported clades in the tree, colored strips indicate their taxonomic distribution (top right legend: Api, Apicomplexa; Euk, Eukaryota; Eug, Euglenozoa; Ubi, ubiquitous; Bab, Babesia; The, Theileria; Fun, Fungi; Tox, Toxoplasma; Eim, Eimeria). The apicomplexan-specific GNA1 family is enclosed within a continuous line box, while the HAT and the NMT families are enclosed within a dashed line box. A schematic protein architecture delineating the occurrence of conserved motifs detected using MEME is shown next to each protein (see Table S2). The distance between vertical lines on the right is 200 amino acids.

To identify apicomplexan proteins presenting a GNAT domain, we searched the genomes of *P. falciparum* and 6 additional species, representing the main lineages of the phylum, for sequences containing the INTERPRO protein functional domain IPR000182 (GNAT domain). 71 protein sequences were detected, belonging to 18 orthogroups plus one singleton in the EupathDB database (http://eupathdb.org/eupathdb/, Supplementary Tables S1 and S2). In order to reconstruct the evolutionary relationships between these sequences, we performed phylogenetic analyses using three alternative methods (see Methods). 9 out of the 18 orthogroups, encompassing 48 out of the 71 sequences, were retrieved by all three methods as well-supported clades in the tree (Fig. 2). Despite the overall high degree of divergence in terms of sequence composition and length, the protein domain architecture within each of these 9 clades appeared relatively well-conserved. Many of the non-GNAT domains in these clades matched INTERPRO protein functional domains generically associated to the GNAT domain, such as the Acyl-CoA *N*-acyltransferase domain, or associated to specific families of GNAT proteins, such as *N*-myristoyltransferases (NMT) or histone acetyltransferases (HAT) (Supplementary Table S3). The remaining 9 orthogroups, clustering 23 out of 71 sequences, were not properly resolved in our phylogenetic analyses. A possible reason for this may be related to the unusually long branches recovered for some *T. gondii* sequences, which may lead to unexpected clustering in the tree^37^. *T. gondii* showed 15 GNAT-domain containing proteins compared to 9–11 in the remaining species, likely resulting from lineage-specific gene duplications (Fig. 2; Supplementary Table S1).

### Identification of the GNA1 family in Apicomplexa

In yeast and other eukaryotic organisms, the glucosamine-6-phosphate acetyltransferase activity is known to be essential and encoded by GNA1, a specific family of GNAT proteins^26,27,33^. The evolutionarily conserved GNA1 gene family typically encodes proteins of 150–200 aa length and only displays the GNAT and/or Acyl-CoA *N*-acyltransferase domains^32^. Out of the ten *P. falciparum* GNAT sequences, five were discarded as candidate GNA1s as they also contained protein domains corresponding to specific families of GNAT proteins other than GNA1. The remaining five (Fig. 2) were selected as candidate sequences and assayed for their putative GNA1 activity.

**Figure 3:**
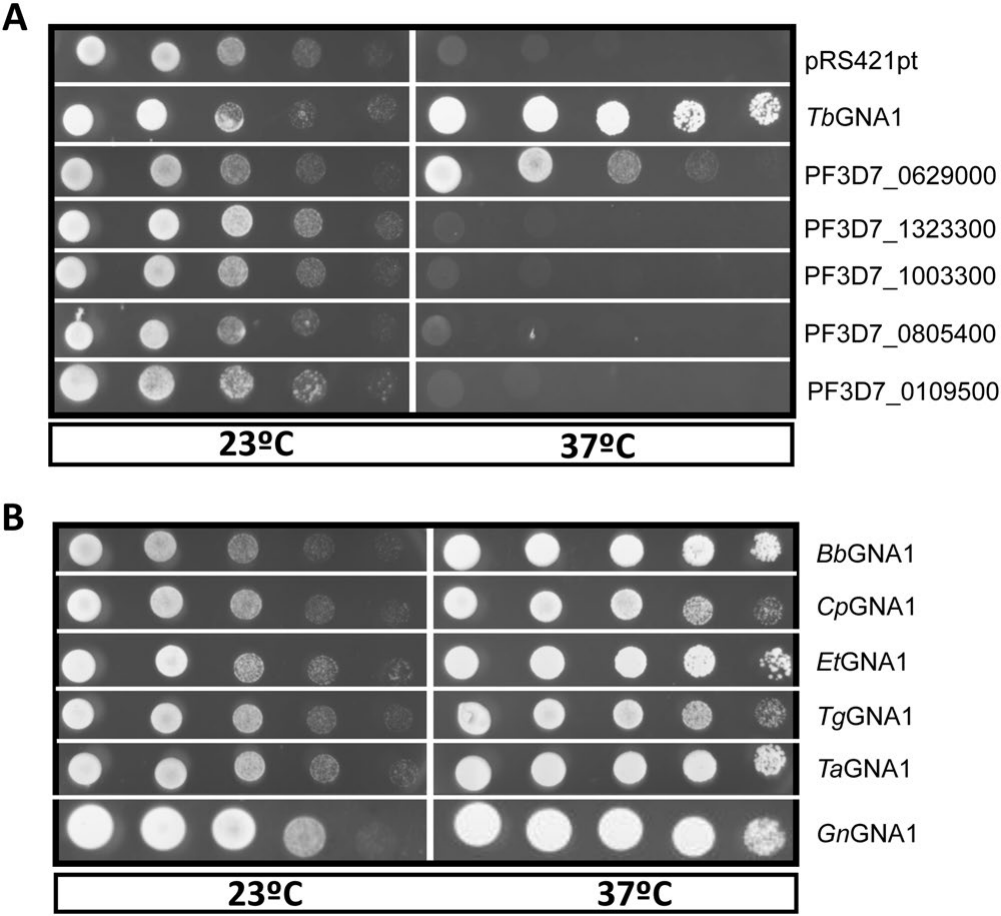
Complementation of *Sc*GNA1-ts by *Pf*GNA1 and other Apicomplexa orthologs. (**A**) *S. cerevisiae* GNA1 thermosensitive mutants GNA1-ts transformed with one of five *P. falciparum* candidate sequences, *Trypanosoma brucei* GNA1 (*Tb*GNA1) or an empty MET15 vector (pRS421 pt), were grown in serial dilution on minimal media without uracil, methionine and cysteine (MM -ura -met -cys) at 23 °C and 37 °C. (**B**) GNA1-ts cells containing GNA1 orthologous sequences from representative apicomplexan species were cloned in pRS421 pt and grown in serial dilution on MM -ura -met -cys at 23 °C and 37 °C. *Bb*, *Babesia bovis*; *Cp*, *Cryptosporidium parvum*; *Et*, *Eimeria tenella*; *Tg*, *Toxoplasma gondii*; *Ta*, *Theileria annulata*; and *Gn*, *Gregarina niphandrodes*.

The five selected *P. falciparum* candidate sequences and *Trypanosoma brucei* GNA1 (*Tb*GNA1)^26^ were cloned into the pRS421 pt plasmid and used to transform the *S. cerevisiae* GNA1 thermosensitive (*Sc*GNA1-ts) mutant^38^. Only PF3D7_0629000 and *Tb*GNA1 could support yeast growth in the absence of endogenous GNA1 at 37 °C (Fig. 3A), and we consequently renamed PF3D7_0629000 as *Pf*GNA1. Our phylogenetic analyses (Fig. 2) clustered *Pf*GNA1 together with six additional sequences within a robustly supported clade corresponding to orthogroup OG5_147324. The predicted orthologs to *Pf*GNA1 from *Babesia bovis*, *C. parvum*, *Eimeria tenella*, *T. gondii*, *Theileria annulata*, and *G. niphandrodes* were also cloned in pRS421 pt and functional complementation assays were performed. All orthologous sequences were able to rescue the *Sc*GNA1-ts mutant at 37 °C (Fig. 3B).

### Independent evolutionary origin of the Apicomplexa-specific GNA1 family

**Figure 4:**
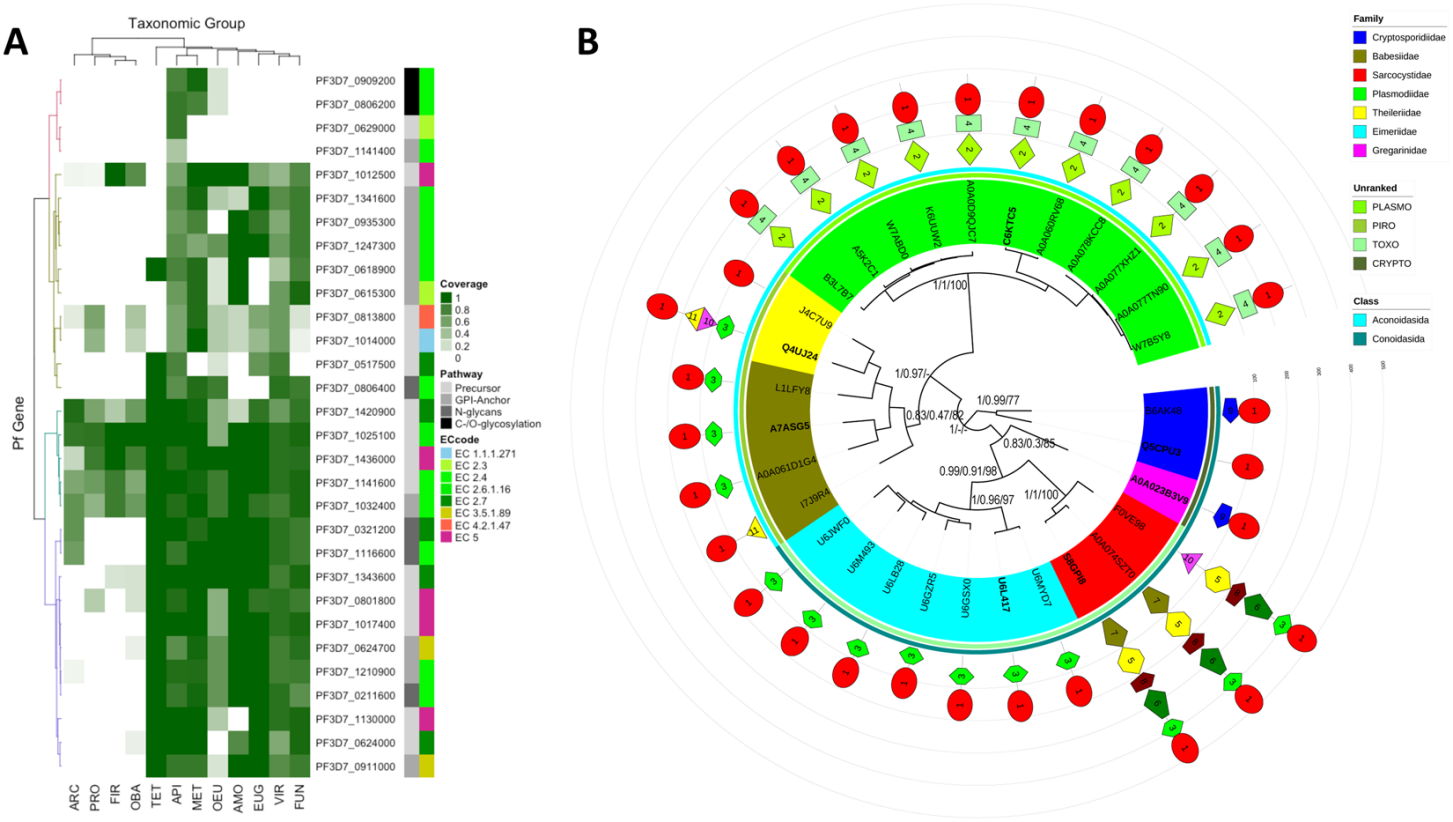
Independent evolutionary origin of Apicomplexa GNA1 family. (**A**) The heat map represents the taxonomic distribution for 30 sugar nucleotide enzyme genes present in *P. falciparum*, i.e., the fraction of 150 species from 12 eukaryote and prokaryote lineages containing orthologs of this gene within each taxonomic lineage, as inferred from the information provided in OrthoMCLDB. Orthogroups have been hierarchically clustered into four clusters based on the Euclidean distance between their taxonomic coverages^62,67^. The biochemical pathways they are involved in and the EC nomenclature of the enzymatic reaction catalyzed are indicated as colored side bars. Fir, Firmicutes; Pro, Proteobacteria; Oba, Other bacteria; Arc, Archaea; Eug, Euglenozoa; Amo, Amoebozoa; Vir, Viridiplantae; Tet, Tetrahymena thermophila; Api, Apicomplexa; Fun, Fungi; Met, Metazoa; Oeu, Other eukaryotes. See also see Table S4. (**B**) Phylogeny and protein domain architecture of putative GNA1s from 30 apicomplexan species. The Bayesian circular phylogenetic tree is drawn to scale, with branch lengths proportional to evolutionary distances between nodes. The tree has been rooted using Cryptosporidiidae representatives, considered to be occupying a basal position among Apicomplexa^41^. Values next to relevant nodes/clades indicate statistical support from maximum-likelihood, Bayesian and neighbor-joining phylogenetic analysis, respectively. Leaves are colored according to their taxonomic family membership and two additional classification groups, including class (see Table S5). Sequences examined for GNA1 activity are indicated in bold. The architecture in terms of conserved protein motifs detected using MEME is shown next to each protein (see Table S6). Outer circular lines form a protein length scale, the distance between lines is 100 amino acids.

According to the OrthoMCLDB database, of all orthogroups grouping Apicomplexa GNAT proteins, only OG5_144531 and OG5_147324 (Apicomplexa GNA1 family) showed a taxonomic distribution restricted to apicomplexan organisms (Supplementary Table S2), suggesting their independent evolutionary origin within the phylum. In order to examine whether this represents an unusual feature of the Apicomplexa GNA1 family, we used the information provided by OrthoMCLDB to look at the taxonomic distribution of 30 *P. falciparum*^29,34^ enzymes involved in sugar nucleotide biosynthesis and related pathways across 150 species representing 12 eukaryote and prokaryote lineages (Fig. 4A, Supplementary Table S4). For each associated orthogroup, we plotted as a heat map the fraction of species within each taxonomic lineage represented by at least one orthologous sequence. The orthogroup containing Apicomplexa GNA1s clusters together with the presumed subunit H of phosphatidylinositol *N*-acetylglucosaminyltransferase (PIG-A subunit H, PF3D7_1141400), involved in GPI-anchor biosynthesis^39^. These two were the only orthogroups with no potential orthologs outside of Apicomplexa, including *Tetrahymena termophila*, belonging to Ciliophora, the sister phylum to Apicomplexa.

Moreover, using simple reciprocal best-hit BLAST searches between *P. falciparum* and human (E < 10^−5^), no potential human orthologs could be unambiguously defined for only two of the 30 *P. falciparum* enzymes, including *Pf*GNA1 and PF3D7_0517500 (Supplementary Table S4). All together these analyses supported a rather exceptional evolutionary history of the Apicomplexa GNA1 family, compared to that of other enzyme families involved in glycoconjugate synthesis.

To further substantiate the orthologous relationships of genes belonging to the Apicomplexa GNA1 family, we examined their syntenic arrangements. A pairwise synteny analysis was performed by comparing the genomic region containing *Pf*GNA1 in *P. falciparum* and 10 additional *Plasmodium* species, revealing series of collinear genes between the two regions (Supplementary Fig. S1). Similarly, synteny was examined both within and between species belonging to Aconoidasida (*P. falciparum* and *B. bovis*) and Conoidasida (*C. parvum*, *T. gondii and T. annulata*), the two main classes in the phylum (Supplementary Fig. S2). Despite the deep evolutionary divergence between the two classes within the phylum (~817 MYA)^40^, synteny could also be observed in some pairwise comparisons along hundreds of Mb of genomic regions, further supporting the single and independent origin of the GNA1 family in Apicomplexa.

We also used the alignment of the GNAT protein domain conserved regions (resulting after removing poorly aligned or highly diverged regions) from the seven Apicomplexa GNA1 family sequences in Fig. 2 as a seed to build a Hidden Markov Model (HMM) profile. The resulting HMM profile was in turn used as a query in iterative searches against the UNIPROT database, with the HMM profile being iteratively rebuilt on the basis of the retrieved hits, ultimately resulting in the identification of a set of 50 significant protein hits exclusive to 50 strains from 30 apicomplexan species. We selected a representative strain from each species, including the seven for which GNA1 activity was confirmed experimentally, to perform phylogenetic and sequence analyses (Supplementary Tables S5 and S6). Extensive diversification could be observed among lineages at the level of sequence length and architecture of protein motifs, reflecting the old age of the Apicomplexa clade^40^ (Fig. 4B). However, the phylogenetic relationships among sequences in the tree generally correspond well to the accepted taxonomic relationships among the species represented^41^. This observed congruence between the gene and the species trees is also compatible with a single evolutionary origin of the gene family.

### Comparative sequence analysis between apicomplexan and non-apicomplexan GNA1 families further supports their independent evolutionary origin

The large superfamily of GNAT-domain containing proteins groups different families of enzymes that use acetyl coenzyme A (AcCoA) to transfer an acetyl group to a substrate^30,31^. Subsequent evolutionary diversification of the superfamily would have been shaped by extensive sequence divergence and lineage-specific domain gain and loss. Although highly divergent at the sequence level, GNAT domains are well-conserved in structure and catalytic mechanism^26,32,33^, suggesting a common evolutionary origin. In order to search for signatures of remote amino acid sequence homology between GNAT domains of the Apicomplexa GNA1 and non-apicomplexan eukaryote GNA1 enzyme families, we compared the multiple sequence alignment of the conserved region of the GNAT domain in a dataset of 30 apicomplexan GNA1 sequences to the conserved regions of the GNAT domain from 30 GNA1 from non-apicomplexan eukaryote organisms, ranging from yeast to human (Supplementary Table S7). The conserved regions of the GNAT domain were extracted by removing poorly aligned or highly diverged regions from their respective alignments. The average percentage of pairwise sequence identity within the ca. 90 amino acid length alignment of apicomplexan GNA1s was 50.46% (similarity 67.71%), close to the ones observed within non-apicomplexan GNA1s (49.74%; similarity: 66.97%). In contrast, when apicomplexan and non-apicomplexan GNA1 sequences were aligned together (Supplementary Fig. S3), the average percentage of pairwise sequence identity between the two sets at the level of the conserved region of the GNAT domain drops to 18.06% (similarity 26.49%). The alignment algorithm introduced a gap of 14 aa length in apicomplexan GNA1s, mostly corresponding to the B3 strand of the canonical motif D of the GNAT domain^31^.

**Figure 5:**
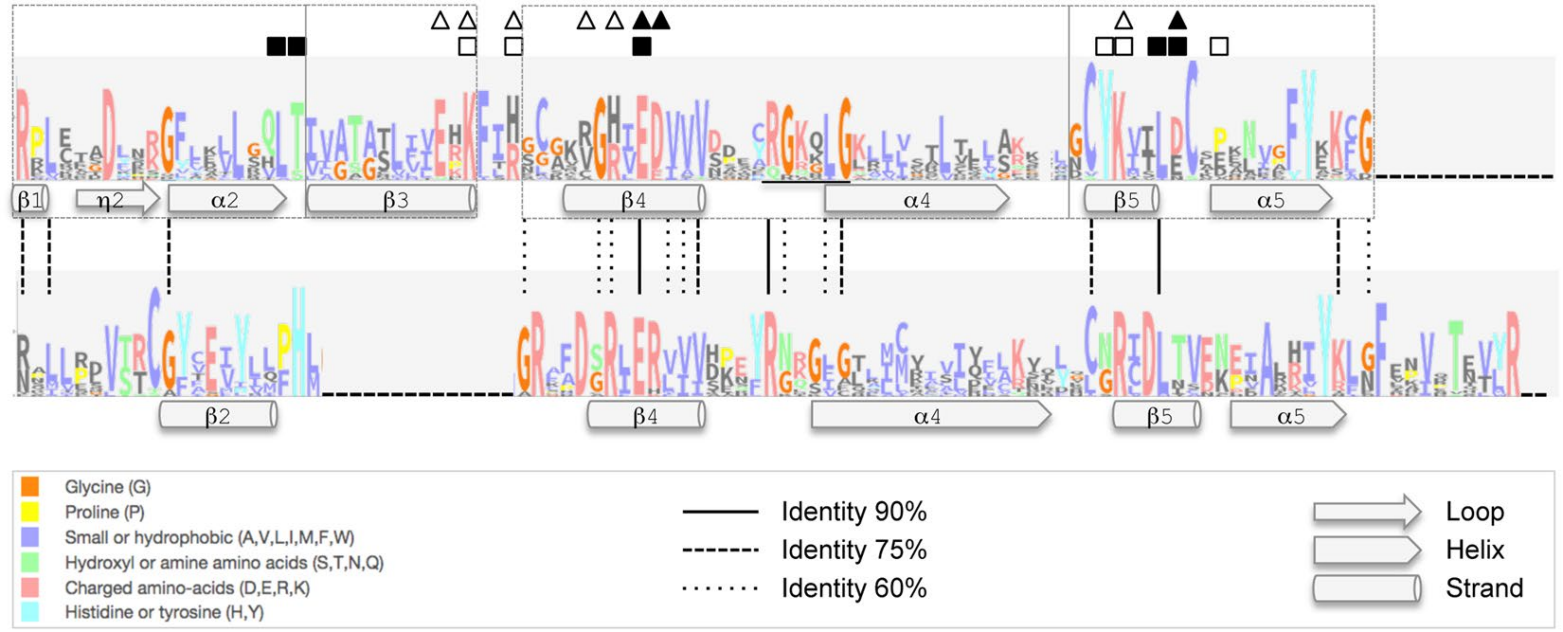
Sequence and secondary structure of the conserved regions of the GNAT domain in apicomplexan and non-apicomplexan eukaryote GNA1s. Logos were generated on the basis of multiple aa sequence alignments of 30 non-apicomplexan eukaryote GNA1s (upper) and 30 apicomplexan GNA1s (lower) resulting after removing poorly aligned or highly diverged regions. Stack height indicates the information content for each position in the alignment, divided by the estimated probability. The residues are colored according to the ClustalX coloring conservation scheme. Vertical lines connect positions conserved in more than 60, 75 or 90% of sequences in the alignments. Regions corresponding to GNAT motifs A-D of the non-apicomplexan eukaryote GNA1 proteins are enclosed within dashed boxes. Functional key residues and secondary structure as determined by the crystal structure of human glucosamine-6-phosphate *N*-acetyltransferase at 2.7 Å resolution (*Hs*GNA1; pdb id: 3cxs) are indicated on the alignment profiles. Residues involved in binding GlcN-6P are shown as squares, and those contributing to the charge distribution as triangles; residues in subunit 1 are shown as filled symbols and those from the other subunit as unfilled symbols. The predicted protein secondary structure for *Pf*GNA1 is also shown. The location of the consensus motif for the GNC5-related *N*-acetyltransferase family is underlined.

18 aa positions in the joint alignment were found to be conserved between apicomplexan and non-apicomplexan GNA1s across more than 60% of the sequences (including seven and three positions conserved in more than 75% and 90% of the sequences, respectively; Fig. 5 and Supplementary Fig. S3). Most of the conserved positions were found within the AcCoA binding motif A, particularly around the consensus motif defined for GCN5-related *N*-acetyltransferases^31^. Notably, only two of the conserved positions correspond to key functional residues identified in the crystal structure of human GNA1 (Fig. 5)^33^.

Lastly, we examined the predicted secondary structure of *Pf*GNA1 and other apicomplexan GNA1 proteins. The region corresponding to the GNAT domain of *Pf*GNA1 showed the characteristic α/β fold^31^, a conformation that was conserved across the remaining six apicomplexan GNA1s examined here (Supplementary Fig. S4). When compared to the secondary structure of the GNAT domain of human GNA1, obtained from its protein crystal structure, some degree of conservation at the structural level could be observed, in particular in motifs A and B at the C-terminal end of the domain. These motifs are involved in acetyl-CoA binding and the active site of the enzyme, respectively^30,31^.

### *In vitro* activity assays of Apicomplexa GNA1 purified proteins confirm GNA1 enzymatic activity

**Figure 6:**
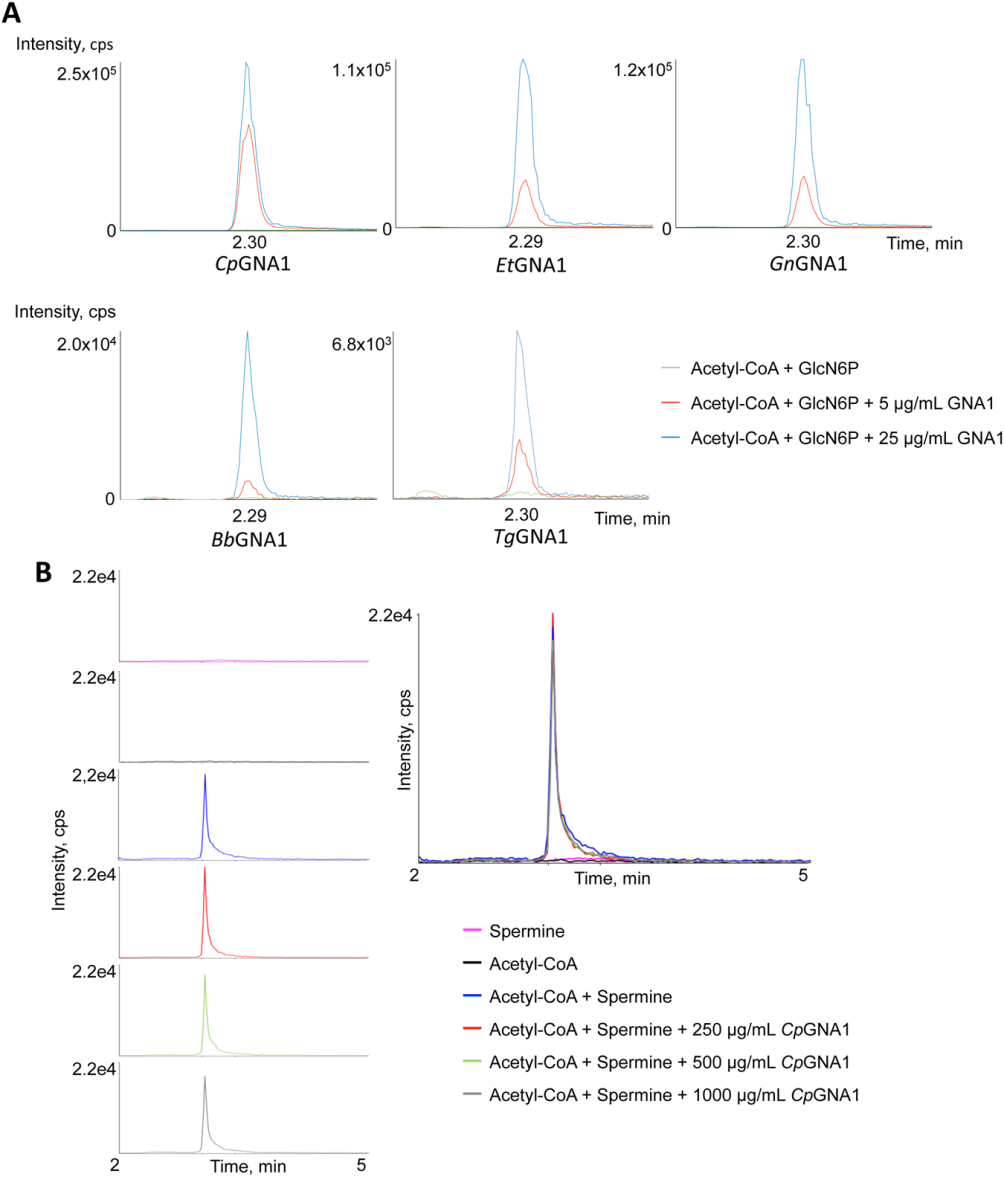
Enzymatic activities of recombinant Apicomplexa GNA1 proteins. (**A**) The purified recombinant GNA1 enzymes were assayed in the presence of GlcN-6P and AcCoA (blue line, 25 µg/mL GNA1; red line, 5 µg/mLGNA1; green line, no GNA1). (**B**) The purified recombinant *Cp*GNA1 enzyme was assayed in the presence of spermine and AcCoA. Chromatograms (left panels) show the detection of an *N*-acetylspermine peak by HPLC-MS/MS in samples containing spermine (pink); AcCoA (black); AcCoA and spermine (blue); AcCoA, spermine and 250 µg/mL of *Cp*GNA1 (red); AcCoA, spermine and 500 µg/mL of *Cp*GNA1 (green); and AcCoA, spermine and 1000 µg/mL of *Cp*GNA1 (grey). Nonenzymatic acetylation^68^ is observed in all the assays containing spermine and AcCoA. In these reactions, the generation of *N*-acetylspermine is not dependent on the concentration of *Cp*GNA1, as it is shown in superimposed chromatograms (right panel).

To further confirm the GNA1 activity of orthologs from the Apicomplexa GNA1 family, we heterologously expressed and purified the corresponding proteins. The enzymes used GlcN-6P as substrate and AcCoA as donor to produce GlcNAc-6P, as observed by HPLC-MS/MS (Fig. 6A). Although *Pf*GNA1 and *Ta*GNA1 were deficiently expressed, they also showed residual activity as detected by HPLC-MS/MS (Supplementary Fig. S5). In addition, the remaining recombinant proteins demonstrated clear GNA1 activity in colorimetric assays (Supplementary Fig. S6).

Since *Cp*GNA1 (cgd4_4000) has been previously described as a spermidine/spermine *N*^1^-acetyltransferase (SSAT), involved in the back-conversion of polyamine spermine to spermidine and putrescine in *C. parvum*^42^, we decided to further characterize its enzymatic activity. Kinetic analyses showed that *Cp*GNA1 used GlcN-6P as substrate to generate GlcNAc-6P displaying classical Michaelis-Menten kinetics, with a K_m_ for AcCoA of 241.7 ± 35.3 µM and 719.1 ± 119.0 µM for GlcN-6P (Fig. S7A). In addition, in our hands *Cp*GNA1 was not able to acetylate spermine or spermidine and SSAT activity could not be detected either in colorimetric (Supplementary Fig. S7B) or HPLC-MS/MS based assays (Fig. 6B). Furthermore, on the basis of the relative levels of GlcNAc-6P generated, *Cp*GNA1 activity was not inhibited by the presence of increasing concentrations of spermine or spermidine (Supplementary Fig. S7C). Hence, our data clearly indicates that the primary biochemical function of *Cp*GNA1 is the acetylation of GlcN-6P. Apparently, *T. gondii* also shows an active polyamine retroconversion metabolism, driven by a SSAT activity, although to our knowledge this enzyme has never been heterologously expressed and assayed *in vitro*^43^. Nevertheless, as in the case of *Cp*GNA1, *Tg*GNA1 distinctly acetylated GlcN-6P, and this activity was neither inhibited by spermine nor spermidine, suggesting that the function of *Tg*GNA1 is indeed to acetylate GlcN-6P (Supplementary Fig. S7C).

### CRISPR-Cas9-based gene disruption strongly suggests *Pf*GNA1 is essential for parasite growth

**Figure 7:**
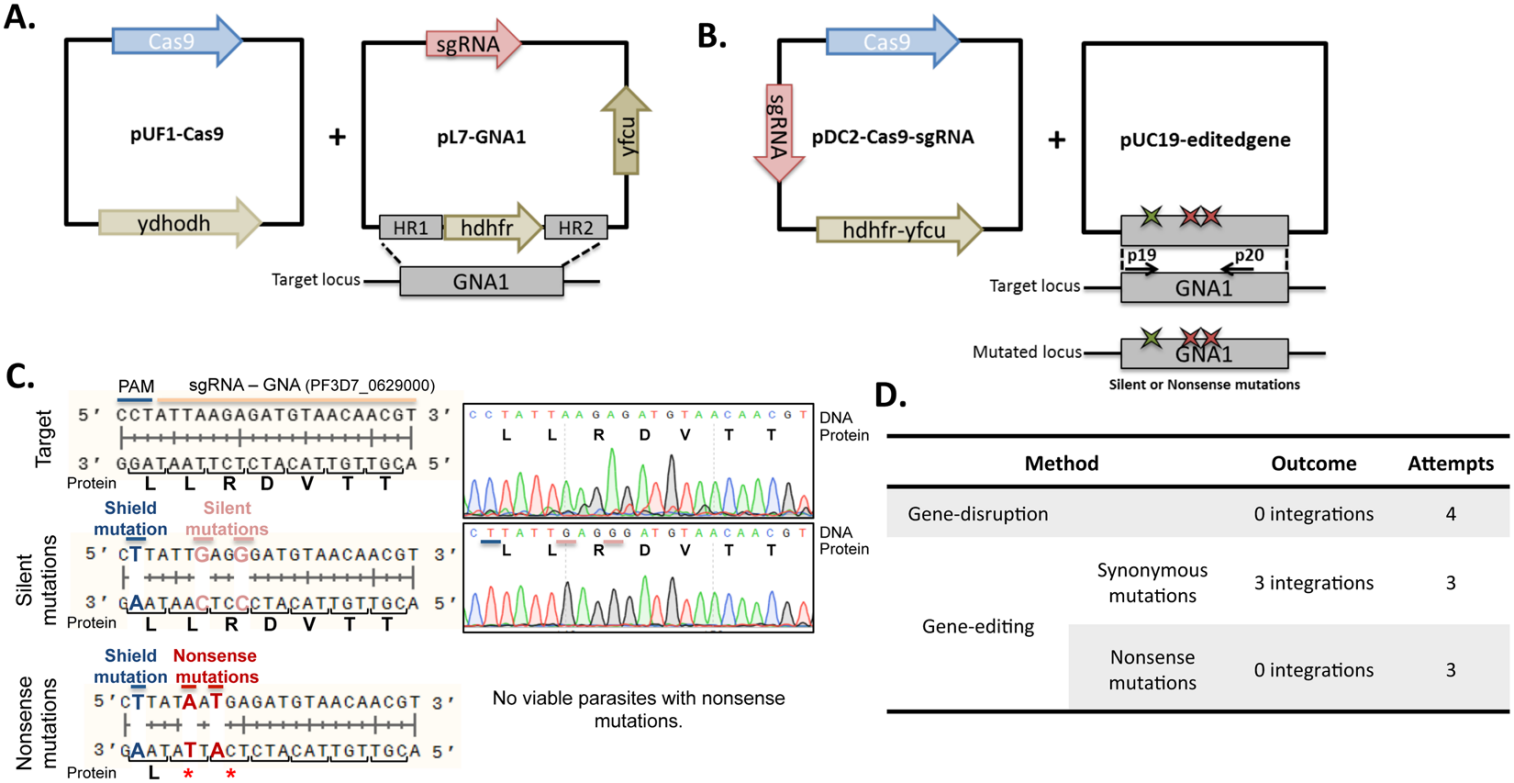
Genetic strategies to disrupt *Pf*GNA1 function. (**A**) Diagram illustrating the strategy used for *Pf*GNA1-disruption. Cas9 protein is expressed by the pUF1-Cas9 episome. pL7-GNA1 episome is continuously maintained using the h*dhfr* selection and carries simultaneously the donor DNA (homology regions: HR1/HR2) and the sgRNA targeting *Pf*GNA1 (pink). (**B**) Diagram illustrating the strategy for *Pf*GNA1 edition. pDC2-Cas9-sgRNA plasmid carries the Cas9 protein and the sgRNA *Pf*GNA1-targeting sequence together with the h*dhfr* and y*fcu* fusion genes for positive and negative selection, respectively. pUC19-edited gene plasmids carry the *Pf*GNA1 donor sequence with shield (green star) and silent or nonsense mutations (red stars) in the sgRNA region generating pUC19-silent*Pf*GNA1 or pUC19-nonsense*Pf*GNA1, respectively. (**C**) Target sequence recognized by *Pf*GNA1 sgRNA and chromatogram of wild-type parasites (top panel), and modified locus sequences and chromatogram of the edited parasites showing the shield (blue) and silent mutations (pink) (middle panel) and nonsense mutations (red) (bottom panel). No parasites were obtained carrying *Pf*GNA1 nonsense mutations. (**D**) Table summarizing *Pf*GNA1 disruption attempts.

Finally, to gain further insight into the biological function of *Pf*GNA1, and to evaluate its potential as a drug target, we assessed whether it was required for parasite growth using CRISPR-Cas9-based techniques. In a first set of experiments, we attempted to disrupt *Pf*GNA1 by CRISPR-Cas9-assisted gene truncation. Whereas controls using analogous plasmids targeting other regions generated viable parasites, no parasites harbouring a truncated version of *Pf*GNA1 could be obtained (Fig. 7A). This suggests that the *Pf*GNA1 gene plays an essential role for the survival of the parasite. In a second set of experiments, we were also unable to introduce nonsense mutations in the N-terminus of the GNAT domain conserved region. In contrast, in three independent biological replicates carried out in parallel, we successfully managed to obtain viable parasites harbouring synonymous nucleotide substitutions in the same region of the GNAT conserved domain (Fig. 7C). Therefore, nucleotides in the catalytic GNAT domain of *Pf*GNA1 are genetically modifiable as long as the open reading frame is not altered. These data strongly suggest that an unaltered version of the *Pf*GNA1 protein is required for parasite viability, at least during *P. falciparum* asexual blood stages.

## Discussion

Although GNA1 enzymes have been identified and characterized throughout the eukaryote kingdom, sequence similarity-based approaches failed to identify GNA1 genes in Apicomplexa^34^. This difficulty was likely due to the extensive divergence at the sequence level featuring the different GNAT domain-containing enzyme families with specific acetyl acceptor substrates and taxonomic coverages. Here, we performed a systematic classification of the large superfamily of GNAT domain-containing proteins in seven Apicomplexa species with fully sequenced genomes^30,31^. Our phylogenetic analysis revealed 9 well-supported gene families displaying conserved architecture of protein motifs. Among them, we identified protein families containing NMT or HAT domains, including representatives of the latter family from *P. falciparum* (PF3D7_0823300) and *T. gondii* (TGGT1_254555 and TGGT1_243440) that had been previously characterized^44,45^. We tested five *P. falciparum* sequences, showing no evident correspondence with other GNAT domain-containing gene families, by complementation assays using a yeast thermosensitive strain defective in GNA1. As a result, we identified *Pf*GNA1 as able to support yeast growth at restrictive temperature. *Pf*GNA1 belongs to a well-defined family containing putative orthologs in all six other Apicomplexa species, all of which rescued the mutant under non-permissive conditions. The GNA1 family in Apicomplexa appears to have had a single origin early during diversification of the phylum, an observation supported by synteny-based analysis. Furthermore, using a HMM profile based on Apicomplexa GNA1 sequences as a query to scan the UNIPROT database, only sequences belonging to the phylum resulted in significant hits. Consistently with a separate evolutionary origin of the family, the phylogenetic tree of putative GNA1 from 30 species of Apicomplexa essentially recapitulated the accepted evolutionary relationships among the species, based on both ultrastructural and developmental characterizations as well as on molecular phylogenies^41^. Altogether, our results support an independent origin of the GNA1 family in Apicomplexa, which can be traced back to between 817 MYA (estimated time of divergence between the two main classes of the phylum) and 1344 MYA (estimated time of divergence between Apicomplexa and its sister group of ciliates represented by *T. termophila*)^40^.

The comparison of the GNAT domains of apicomplexan and non-apicomplexan GNA1s showed that domain conservation was essentially restricted to a few specific amino acid positions around the GNAT six-amino acid consensus motif^31^. Furthermore, the conserved residues were, with very few exceptions, distinct from the ones reported as critical for the catalytic activity of human GNA1^32^. This observation suggests that, rather than evolving from an ancestral eukaryote GNA1 family, apicomplexan GNA1s might have evolved in parallel from a separate GNAT domain-containing gene lineage. Furthermore, considering the evolutionary time since divergence of the phylum from other eukaryotic lineages^40^, Apicomplexa-specific GNA1s may have evolved functionally divergent catalytic residues exploitable for the design of selective small-molecule inhibitors.

The *Cp*GNA1 sequence (cgd4_4000) had been previously characterized to encode a SSAT^42^, involved in the reverse polyamine biosynthetic pathway in *C. parvum*. Here, whereas a robust GNA1 activity could be detected with as low as 0.2 µg/mL of purified *C. parvum* protein, we did not detect a SSAT activity with up to 1000 µg/mL, both through colorimetric and HPLC-MS/MS based *in vitro* assays. In addition, neither *Cp*GNA1 nor *Tg*GNA1^43^ activities were inhibited by the presence of spermine or spermidine. Considering that the rest of the apicomplexan proteins tested showed evident GNA1 activities, our results suggest that the primary function of this family of enzymes is the acetylation of GlcN6P. Thus, the SSAT activity previously described for *Cp*GNA1 would be residual, secondary, and/or just restricted to specific taxonomic lineages, life cycle stages or physiological conditions.

Our multiple efforts to ablate *Pf*GNA1 using CRISPR-Cas9-based techniques were unsuccessful, despite evidence that the locus was genetically modifiable. This strongly suggests that the gene is essential for the growth of the malaria parasite, in agreement with UDP-GlcNAc being a bottleneck metabolite^46^, likely due to its involvement in the biosynthesis of important glycoconjugates, such as GPI anchors. Nevertheless, the relevance of GNA1 in other apicomplexan organisms is at present unknown. Notably, in a recent work, a loss-of-function genome-wide screen was performed in *T. gondii* tachyzoites to assess the contribution of targeted genes to cell fitness^47^. With the exception of *Tg*GNA1 (TGGT1_243600), all the genes putatively encoding for enzymatic activities involved in UDP-GlcNAc biosynthesis (*i.e*. glucosamine:fructose-6-P-amidotransferase, TGGT1_231350; and *N*-acetylglucosamine-phosphomutase, TGGT1_264650) or UDP-GlcNAc utilization (*i.e*. Phosphatidylinositol *N*-acetylglucosaminyltransferase, TGGT1_241860; and *N*-acetylglucosaminyl phosphate transferase, TGGT1_244520) contributed greatly to *T. gondii* survival. This underlines the importance of the amino sugar biosynthetic route, whose presence is confirmed by labelling the glycoproteins of the parasite with [^3^H]GlcN^48,49^. The striking lack of contribution of *Tg*GNA1 to *T. gondii* fitness^47^ might be suggesting the existence of another gene in the parasite’s genome encoding for a redundant GNA1 activity, although it might also be highlighting certain limitations of the aforementioned study, such as the effect on proteins with slow turnover or the time selected for the screening readout^47^. Indeed, considering the comprehensive analysis of GNAT-containing sequences in different Apicomplexa presented here, and the functional complementation assays performed with additional *P. falciparum* sequences, no other suitable candidate sequence encoding for a redundant GNA1 activity could be identified. The generation of conditional GNA1 mutants, still a challenging task in apicomplexan organisms, will contribute to comprehensively define whether GNA1 biochemical activity is essential in different species and to confirm its suitability as a potential therapeutic target for drug development.

## Methods

### Sequence and phylogenetic analysis

Phylogenetic analyses were performed on the basis of multiple alignments of amino acid sequences obtained using MUSCLE^50^. Bayesian and maximum-likelihood analyses were carried out using the Blosum62 + G (eight categories, shape parameter: 1.87) + F protein evolution model^51^, selected by ProtTest v3.2 as the best-fitting to the data^52^. Bayesian analysis was implemented in MrBayes 3.2.5^53^. Searches were run with four Markov chains for one million generations sampling every 100th tree. After the stationary phase was reached, determined by the average standard deviation of split sequences approaching 0 (<0.05), the first 2,500 trees were discarded as burn-in. A consensus tree was then constructed to evaluate posterior probabilities on clades. Maximum-likelihood trees were constructed using PhyML v3.1, with tree topology searching optimized using the subtree pruning and regrafting option^54^. The statistical support of the retrieved topology was assessed using the Shimodaira-Hasegawa-like approximate likelihood ratio test^55^. Neighbor-joining phylogenetic analyses were conducted in Seaview v4.5.4^56^, with statistical support on clades assessed using a bootstrap analysis with 1,000 replicates. Trees were represented and edited using iTOL v3.3.2^57^. MEME v4.11.2 was used to identify conserved motifs shared among proteins^58^. Profile hidden Markov models (HMMs) were generated and calibrated using HMMER v3.0^59^, on the basis of MUSCLE protein alignments^50^ further edited with Gblocks^60^. Logo representations of multiple protein sequence alignments were obtained using Skylign^61^. Protein secondary structures for apicomplexan GNA1 sequences were predicted using SABLE^62^.

### Cloning of apicomplexan GNA1 candidate sequences

pRS421 vector (ATCC 87475), containing ampicillin and *MET15*^63^, was modified by cloning *S. cerevisiae* GPD promoter and CYC1 terminator sequences using SacII/BamHI and XhoI/KpnI restriction sites. The new vector generated, pRS421 pt was used to clone different GNA1 candidate sequences. Genomic DNAs of *C. parvum*, *E. tenella*, *T. gondii*, and *T. brucei* were used as templates for PCR amplification with Platinum DNA Polymerase High Fidelity (Thermo Scientific), using specific primers including BamHI and XhoI restriction sites (Supplementary Table S8). The remaining sequences cloned in pRS421 pt were codon-optimized synthetic genes (GenScript or Integrated DNA Technologies) based on the predicted amino acid sequences.

### Yeast complementation assays

A *S. cerevisiae* GNA1 thermosensitive mutant, *Sc*GNA1-ts^38^, was used for GNA1 complementation assays. The mutant contains a temperature-sensitive copy of *GNA1* (YFL017C), which encodes a GNA1 enzymatic activity essential for yeast survival. The thermosensitive allele is marked by *URA3* and the mutant was not able to grow above 34 °C^38^. *Sc*GNA1-ts contains also a mutated *MET15* gene that allows auxotrophic complementation with pRS421 pt vector in minimal media without uracil, methionine and cysteine (-ura, -met, -cys). After transformation with pRS421 pt based constructs, yeast cells were grown in serial dilution at permissive (23 °C) and restrictive (37 °C) temperatures.

### Apicomplexan GNA1 protein expression and purification

Codon-optimized versions of apicomplexan GNA1 (*Pf*GNA1*, Bb*GNA1, *Cp*GNA1, *Et*GNA1, *Tg*GNA1, *Ta*GNA1, and *Gn*GNA1) native sequences were cloned in a pGEX 6P-1 vector containing an N-terminal glutathione *S*-transferase (GST) tag and transformed in *E. coli* BL21 (DE3). Cultures were grown for 16 h at 30 °C after induction, lysed and supernatants containing the glutathione S-transferase (GST)-tagged GNA1 protein were filtered and purified through GSTrap HP 1-ml or GST SpinTrap columns (GE Healthcare). The proteins of interest were then dialyzed (Thermo Scientific Slide-A-Lyzer MINI Dialysis Devices, 10 K MWCO) at 4 °C using Buffer A (50 mM Tris-HCl pH 7.5; 250 mM NaCl) and stored.

### GNA1 and SSAT *in vitro* assays

GNA1 activity was assayed using 500 µM of AcCoA, 500 µM of GlcN-6P and different concentrations of apicomplexan GNA1 in 50 µl of 25 mM Tris-HCl–150 mM NaCl, pH 7.2 solution. To determine SSAT activity the same protocol was used but using as substrate 500 µM of spermine and 3 different *Cp*GNA1 concentrations (250 µg/ml, 500 µg/ml and 1000 µg/ml). For SSAT colorimetric assays 500 µM of spermine or spermidine were used as substrate and 1, 20 or 500 µg/ml of *Cp*GNA1. Reactions were allowed to proceed for 30 min at room temperature before being stopped by boiling for 10 min.

### Liquid chromatography-electrospray ionization-tandem mass spectrometry (LC-MS/MS)

LC-MS/MS analyses were carried out on an UPLC – Acquity system (Waters) coupled by electrospray ionization to an API3000 triple quadrupole LC-MS/MS mass spectrometer (Perkin-Elmer Sciex), using a Kinetex® 2.6 µm HILIC 100 Å column (150 × 4.6 mm, Phenomenex) for Glc-6P/GlcNAc-6P detection, and a an XBridge HILIC 5.0 µm, 130 Å (150 × 4.6 mm, Waters) for spermine/*N*-acetylspermine detection (see Supplementary Methods).

### CRISPR-Cas9 *Pf*GNA1-disruption and *Pf*GNA1-editing constructs

Parasites were cultured and transfected either by electroporating ring-stage parasites or by nucleofection of schizont stages, as previously described^64^. All the methods were carried out in accordance with relevant guidelines and regulations and human erythrocytes and serum were purchased from the Banc de Sang i Teixits (Catalonia, Spain), after ethical approval from the Comitè Ètic Investigació Clínica Hospital Clínic de Barcelona. A single guide RNA (sgRNA) targeting the *Pf*GNA1 consensus motif [(R/Q)-X-X-Q-X-G] was chosen using the Eukaryotic Pathogen CRISPR gRNA Design Tool^65^. For *Pf*GNA1-disruption homology regions (HR) 1 and 2 were amplified from 3D7 *P. falciparum* genomic DNA using primers P1/P2 and P3/P4 and cloned in plasmid pL7 using SpeI/AflII and EcoRI/NcoI restriction sites, respectively^64^. sgRNA was integrated replacing pL7 BtgZI-adaptor (Fig. 7A). For *Pf*GNA1 edition, sgRNA and Cas9-expressing construct (pDC2-Cas9-hDHFRyFCU)^66^ and linearized pUC19 plasmids were used as backbone. The sgRNA sequence was cloned in the pDC2-Cas9-sgRNA using primers P5/P6. *Pf*GNA1 coding sequences with a shield mutation in the protospacer-adjacent motif (PAM)^64^ and silent or nonsense mutations were cloned in pUC19 to generate pUC19-silent *Pf*GNA1 or pUC19-nonsense *Pf*GNA1 (Fig. 7B). All primers used are described in Supplementary Table S8.

## Electronic supplementary material

Supplementary Information

Supplementary Table 1

Supplementary Table 2

Supplementary Table 3

Supplementary Table 4

Supplementary Table 5

Supplementary Table 6

Supplementary Table 7

Supplementary Table 8

## References

[1] Cavalier-Smith T (1993). Kingdom protozoa and its 18 phyla. Microbiol. Rev..

[2] Adl SM (2007). Diversity, Nomenclature, and Taxonomy of Protists. Syst. Biol..

[3] World Health Organization. *World Malaria Report, 2017* (2017).

[4] Torgerson PR, Mastroiacovo P (2013). The global burden of congenital toxoplasmosis: a systematic review. Bull. World Health Organ..

[5] Kotloff KL (2013). Burden and aetiology of diarrhoeal disease in infants and young children in developing countries (the Global Enteric Multicenter Study, GEMS): a prospective, case-control study. Lancet (London, England).

[6] Blake DP, Tomley FM (2014). Securing poultry production from the ever-present Eimeria challenge. Trends Parasitol..

[7] Bock R, Jackson L, de Vos A, Jorgensen W (2004). Babesiosis of cattle. Parasitology.

[8] Schawang JE, Janovy J (2001). The Response of Gregarina niphandrodes (Apicomplexa: Eugregarinida: Septatina) to Host Starvation in Tenebrio molitor (Coleoptera: Tenebrionidae) Adults. J. Parasitol..

[9] Martens C, Vandepoele K (2008). & Van de Peer, Y. Whole-genome analysis reveals molecular innovations and evolutionary transitions in chromalveolate species. Proc. Natl. Acad. Sci. USA.

[10] Coppens I (2013). Targeting lipid biosynthesis and salvage in apicomplexan parasites for improved chemotherapies. Nat. Rev. Microbiol..

[11] Rodrigues JA (2015). Parasite Glycobiology: A Bittersweet Symphony. PLoS Pathog..

[12] McConville MJ, Ferguson MA (1993). The structure, biosynthesis and function of glycosylated phosphatidylinositols in the parasitic protozoa and higher eukaryotes. Biochem. J..

[13] Striepen B (1997). Molecular structure of the ‘low molecular weight antigen’ of Toxoplasma gondii: a glucose α1-4 N-acetylgalactosamine makes free glycosyl-phosphatidylinositols highly immunogenic. J. Mol. Biol..

[14] Naik RS (2000). Glycosylphosphatidylinositol Anchors of Plasmodium falciparum: Molecular Characterization and Naturally Elicited Antibody Response That May Provide Immunity to Malaria Pathogenesis. J. Exp. Med..

[15] Doering TL, Masterson WJ, Englund PT, Hart GW (1989). Biosynthesis of the glycosyl phosphatidylinositol membrane anchor of the trypanosome variant surface glycoprotein. Origin of the non-acetylated glucosamine. J. Biol. Chem..

[16] Bushkin GG (2010). Suggestive evidence for Darwinian Selection against asparagine-linked glycans of Plasmodium falciparum and Toxoplasma gondii. Eukaryot. Cell.

[17] Haserick JR, Leon DR, Samuelson J, Costello CE (2017). Asparagine-linked Glycans of Cryptosporidium parvum Contain a Single Long Arm, Are Barely Processed in the ER or Golgi, and Show a Strong Bias for Sites with Threonine. Mol. Cell. Proteomics.

[18] Burda P, Aebi M (1999). The dolichol pathway of N-linked glycosylation. Biochim. Biophys. Acta.

[19] Samuelson J, Robbins PW (2015). Effects of N-glycan precursor length diversity on quality control of protein folding and on protein glycosylation. Semin. Cell Dev. Biol..

[20] Izquierdo L (2009). Distinct donor and acceptor specificities of Trypanosoma brucei oligosaccharyltransferases. EMBO J..

[21] Parodi AJ (1993). N-glycosylation in trypanosomatid protozoa. Glycobiology.

[22] Banerjee S, Robbins PW, Samuelson J (2008). Molecular characterization of nucleocytosolic O-GlcNAc transferases of Giardia lamblia and Cryptosporidium parvum. Glycobiology.

[23] Cevallos AM (2000). Mediation of Cryptosporidium parvum infection *in vitro* by mucin-like glycoproteins defined by a neutralizing monoclonal antibody. Infect. Immun..

[24] Tomita T (2017). Making Home Sweet and Sturdy: Toxoplasma gondii ppGalNAc-Ts Glycosylate in Hierarchical Order and Confer Cyst Wall Rigidity. MBio.

[25] Riegler H (2012). Crystal structure and functional characterization of a glucosamine-6-phosphate N-acetyltransferase from Arabidopsis thaliana. Biochem. J..

[26] Mariño K (2011). Characterization, localization, essentiality, and high-resolution crystal structure of glucosamine 6-phosphate N-acetyltransferase from Trypanosoma brucei. Eukaryot. Cell.

[27] Mio T, Yamada-Okabe T, Arisawa M, Yamada-Okabe H (1999). Saccharomyces cerevisiae GNA1, an essential gene encoding a novel acetyltransferase involved in UDP-N-acetylglucosamine synthesis. J. Biol. Chem..

[28] Stokes MJ (2008). The synthesis of UDP-N-acetylglucosamine is essential for bloodstream form trypanosoma brucei *in vitro* and *in vivo* and UDP-N-acetylglucosamine starvation reveals a hierarchy in parasite protein glycosylation. J Biol Chem.

[29] Cova M, Rodrigues JA, Smith TK, Izquierdo L (2015). Sugar activation and glycosylation in Plasmodium. Malar. J..

[30] Dyda F, Klein DC, Hickman AB (2000). GCN5-Related N-Acetyltransferases: A Structural Overview. Annu. Rev. Biophys. Biomol. Struct..

[31] Vetting MW (2005). Structure and functions of the GNAT superfamily of acetyltransferases. Arch. Biochem. Biophys..

[32] Wang J, Liu X, Liang Y-H, Li L-F, Su X-D (2008). Acceptor substrate binding revealed by crystal structure of human glucosamine-6-phosphate *N* -acetyltransferase 1. FEBS Lett..

[33] Hurtado-Guerrero R (2008). Structural and kinetic differences between human and *Aspergillus fumigatus* D-glucosamine-6-phosphate *N* -acetyltransferase. Biochem. J..

[34] Sanz S (2013). Biosynthesis of GDP-fucose and other sugar nucleotides in the blood stages of Plasmodium falciparum. J. Biol. Chem..

[35] Sanz S (2016). The disruption of GDP-fucose de novo biosynthesis suggests the presence of a novel fucose-containing glycoconjugate in Plasmodium asexual blood stages. Sci. Rep..

[36] López-Gutiérrez B, Dinglasan RR, Izquierdo L (2017). Sugar nucleotide quantification by liquid chromatography tandem mass spectrometry reveals a distinct profile in *Plasmodium falciparum* sexual stage parasites. Biochem. J..

[37] Felsenstein, J. *Inferring phylogenies*. (Sinauer Associates, 2004).

[38] Ben-Aroya S (2008). Toward a comprehensive temperature-sensitive mutant repository of the essential genes of Saccharomyces cerevisiae. Mol. Cell.

[39] Kinoshita T, Inoue N (2000). Dissecting and manipulating the pathway for glycosylphos-phatidylinositol-anchor biosynthesis. Curr. Opin. Chem. Biol..

[40] Parfrey LW, Lahr DJG, Knoll AH, Katz LA (2011). Estimating the timing of early eukaryotic diversification with multigene molecular clocks. Proc. Natl. Acad. Sci. USA.

[41] Kuo C-H, Wares JP, Kissinger JC (2008). The Apicomplexan whole-genome phylogeny: an analysis of incongruence among gene trees. Mol. Biol. Evol..

[42] Yarlett N (2007). Cryptosporidium parvum spermidine/spermine N1-acetyltransferase exhibits different characteristics from the host enzyme. Mol. Biochem. Parasitol..

[43] Cook T (2007). Divergent polyamine metabolism in the Apicomplexa. Microbiology.

[44] Fan Q, An L, Cui L (2004). Plasmodium falciparum histone acetyltransferase, a yeast GCN5 homologue involved in chromatin remodeling. Eukaryot. Cell.

[45] Bhatti MM, Livingston M, Mullapudi N, Sullivan WJ (2006). Pair of unusual GCN5 histone acetyltransferases and ADA2 homologues in the protozoan parasite Toxoplasma gondii. Eukaryot. Cell.

[46] Chiappino-Pepe A, Tymoshenko S, Ataman M, Soldati-Favre D, Hatzimanikatis V (2017). Bioenergetics-based modeling of Plasmodium falciparum metabolism reveals its essential genes, nutritional requirements, and thermodynamic bottlenecks. PLOS Comput. Biol..

[47] Sidik SM (2016). A Genome-wide CRISPR Screen in Toxoplasma Identifies Essential Apicomplexan Genes. Cell.

[48] Zinecker CF (2001). Two glycoforms are present in the GPI-membrane anchor of the surface antigen 1 (P30) of Toxoplasma gondii. Mol. Biochem. Parasitol..

[49] Tomavo S, Schwarz RT, Dubremetz JF (1989). Evidence for glycosyl-phosphatidylinositol anchoring of Toxoplasma gondii major surface antigens. Mol. Cell. Biol..

[50] Edgar RC (2004). MUSCLE: multiple sequence alignment with high accuracy and high throughput. Nucleic Acids Res..

[51] Henikoff S, Henikoff JG (1992). Amino acid substitution matrices from protein blocks. Proc. Natl. Acad. Sci. USA.

[52] Darriba D, Taboada GL, Doallo R, Posada D (2011). ProtTest 3: fast selection of best-fit models of protein evolution. Bioinformatics.

[53] Ronquist F, Huelsenbeck JP (2003). MrBayes 3: Bayesian phylogenetic inference under mixed models. Bioinformatics.

[54] Guindon S (2010). New algorithms and methods to estimate maximum-likelihood phylogenies: assessing the performance of PhyML 3.0. Syst. Biol..

[55] Anisimova M, Gascuel O (2006). Approximate Likelihood-Ratio Test for Branches: A Fast, Accurate, and Powerful Alternative. Syst. Biol..

[56] Gouy M, Guindon S, Gascuel O (2010). SeaView version 4: A multiplatform graphical user interface for sequence alignment and phylogenetic tree building. Mol. Biol. Evol..

[57] Letunic I, Bork P (2016). Interactive tree of life (iTOL)v3: an online tool for the display and annotation of phylogenetic and other trees. Nucleic Acids Res..

[58] Bailey TL (2009). MEME SUITE: tools for motif discovery and searching. Nucleic Acids Res..

[59] Eddy SR (2011). Accelerated Profile HMM Searches. PLoS Comput. Biol..

[60] Castresana J (2000). Selection of Conserved Blocks from Multiple Alignments for Their Use in Phylogenetic Analysis. Mol. Biol. Evol..

[61] Wheeler TJ, Clements J, Finn RD (2014). Skylign: a tool for creating informative, interactive logos representing sequence alignments and profile hidden Markov models. BMC Bioinformatics.

[62] Adamczak, R., Porollo, A. & Meller, J. SABLE protein structure prediction server at, http://sable.cchmc.org/ (2003).

[63] Baker Brachmann C (1998). Designer Deletion Strains derived from Saccharomyces cerevisiae S288C: a Useful set of Strains and Plasmids for PCR-mediated Gene Disruption and Other Applications. Yeast.

[64] Ghorbal M (2014). Genome editing in the human malaria parasite Plasmodium falciparum using the CRISPR-Cas9 system. Nat. Biotechnol..

[65] Peng D, Tarleton R (2015). EuPaGDT: a web tool tailored to design CRISPR guide RNAs for eukaryotic pathogens. Microb. genomics.

[66] Ng CL (2016). CRISPR-Cas9-modified p*fmdr1* protects *Plasmodium falciparum* asexual blood stages and gametocytes against a class of piperazine-containing compounds but potentiates artemisinin-based combination therapy partner drugs. Mol. Microbiol..

[67] Ward JH (1963). Hierarchical Grouping to Optimize an Objective Function. J. Am. Stat. Assoc..

[68] Wallace, H. M. & Evans, D. M. In *Polyamine Protocols* 59–68 (Humana Press, 1998).

