# Supplementary material for: The Apicomplexa-specific glucosamine-6-phosphate N-acetyltransferase gene family encodes a key enzyme for glycoconjugate synthesis with potential as therapeutic target
Source: Sci Rep. 2018 Mar 5;8:4005. doi: 10.1038/s41598-018-22441-3 (PMC5838249; doi:10.1038/s41598-018-22441-3)
Supplement: Supplementary file 1 — Supplementary Information [file 41598_2018_22441_MOESM1_ESM.pdf]

The Apicomplexa-specific glucosamine-6-phosphate N-acetyltransferase gene family encodes a key enzyme for glycoconjugate synthesis with potential as therapeutic target

M. Cova, B. López-Gutiérrez, S. Artigas-Jerónimo, A. González-Díaz, G. Bandini, S. Maere, L. Carretero-Paulet and L. Izquierdo

Supplementary Information

Supplementary Materials and Methods

Sequence and phylogenetic analysis

Phylogenetic analyses of GNA1s from 30 apicomplexan species were performed on the basis of multiple alignments of amino acid sequences obtained using MUSCLE [1]. Maximum-likelihood and Bayesian analyses were carried out using the JTT protein evolution model [2], with the proportion of invariable sites fixed at 0.041, the heterogeneity of amino acid substitution rates corrected according to a γ-distribution with eight rate categories (G, shape parameter: 1.78), and amino acid frequencies estimated from the data (F). The JTT amino acid substitution model was selected by ProtTest v3.2 as the best-fitting to the data [3]. Bayesian analysis was implemented in MrBayes 3.2.5 [4]. Searches were run with four Markov chains for one million generations sampling every 1000th tree. After the stationary phase was reached, determined by the average standard deviation of split sequences approaching 0 (< 0.05), which reflects the fact that independent tree samples became increasingly similar, the first 250 trees were discarded as burn-in. A consensus tree was then constructed to evaluate Bayesian posterior probabilities on clades. Maximum-likelihood trees were constructed using PhyML v3.1 [5]. Tree topology searching was optimized using the subtree pruning and regrafting option. The statistical support of the retrieved topology was assessed using the Shimodaira-Hasegawa-like approximate likelihood ratio test [6]. Neighbor-joining phylogenetic analyses were conducted in Seaview v4.5.4 [7]. To obtain statistical support on the resulting clades, a bootstrap analysis with 1000 replicates was performed. Resulting trees were represented and edited using the Interactive Tree Of Life (iTOL) v3.3.2 [8].

MEME v4.11.2 was used to identify conserved motifs shared among proteins [9]. The best possible match for each retrieved motif was scanned against the INTERPRO database of protein functional domains. Protein alignments of the conserved regions corresponding to the GNAT domain of three datasets of 30 representative sequences of the Apicomplexa GNA1 and the eukaryote GNA1 protein families, were obtained using MUSCLE [1], and were further edited with Gblocks [10]. Protein secondary structure for Apicomplexa GNA1s was predicted using SABLE [11].

Genomic DNA extractions

Extractions of genomic DNA from *T. gondii* RH tachyzoites, *E. tenella* oocysts and *C. parvum* (Iowa strain) oocysts (purchased from Bunch Frass Farm, Dury, ID) were performed with DNAzol (Life Technologies) according to manufacturer's instructions. For *T. gondii*, tachyzoites were harvested by centrifugation and directly lysed in DNAzol. For *E. tenella,* sporulated oocysts were collected by centrifugation, washed in 1xPBS and resuspended in DNAzol. After adding equal volume of glass beads (425-600 µm, Sigma), the oocysts were vortexed at max speed for 5 min and subjected to 3 freeze/thaw cycles. Samples were examined at an inverted light microscope to determine that > 90% of oocysts were broken. *C. parvum* sporulated oocysts were excysted as described in Banerjee et al. [12] and the resulting sporozoites were lysed in DNAzol. All samples were treated with 10 µg/ml RNaseA for 2 h at 37°C.

Liquid chromatography-electrospray ionization-tandem mass spectrometry (LC-MS/MS)

LC-MS/MS analyses were carried out on an UPLC – Acquity system (Waters) coupled by electrospray ionization to an API3000 triple quadrupole LC-MS/MS mass

spectrometer (Perkin-Elmer Sciex). For Glc-6P/GlcNAc-6P detection, a Kinetex® 2.6 µm HILIC 100 Å column (150 x 4.6 mm, Phenomenex) was used at 40ºC and samples (10 µL from *in vitro* enzymatic assays) were injected. The binary mobile phase was composed of methanol + 0.1% formic acid (A) and 10 mM ammonium formate at pH 3.2 (B) at a constant flow rate of 1200 µL/min. Starting conditions (98% A and 2% B) were maintained for 1 minute followed by a 1 minute gradient to 2% A, which was maintained for 4 minutes. Before running the next sample, the column was re-equilibrated by returning to the initial conditions of 98% methanol + 0.1% formic acid for 2 minutes. The mass spectrometer was operated in the negative ion mode with the following settings: ion spray voltage -4200V, declustering potential (DP) -45V and collision energy (CE) -25V. The ion transitions at *m/z* 258.2⇒97.1 and 300.2⇒97.1 were selected for monitoring GlcN-6P and GlcNAc-6P, respectively. For spermine/*N*-acetylspermine detection, an XBridge HILIC 5.0 µm, 130 Å (150 x 4.6 mm, Waters) column was used, and the binary mobile phases were composed of methanol with 0.1% formic acid (A) and 10 mM ammonium formate at pH 3.2 (B). Starting conditions (98% A) were maintained for 1 minute followed by a 1 minute gradient to 2% A, which was maintained for 4 minutes. Before running the next sample, the column was re-equilibrated by returning to the initial conditions of 98% methanol + 0.1% formic acid for 2 minutes. The flow rate was set at 1200 µL/min and 10 µL from *in vitro* enzymatic assays were injected. The mass spectrometer was operated in the positive ion mode with an ion spray voltage of 4500V. Spermine was monitored using *m/z* 129.1⇒84.1 ion transition, 45V DP and 31.5 CE. *N*-acetylspermine was monitored using *m/z* 100.1⇒72.2 ion transition, 65V DP and 18 CE.

GNA1 and SSAT colorimetric assays

In vitro activity colorimetric assays, based on the detection of Coenzyme A (CoASH), generated during acetyl transfer, by reaction with the thiol reagent 5,5′-Dithiobis(2-nitrobenzoic acid) (DTNB), were adapted from the literature [13,14]. AcCoA was used as acetyl donor and GlcN-6P, spermine or spermidine as substrates (supplied by Sigma). All reactions were carried out at room temperature in triplicate in a 96-well-plate format. To determine GNA1 activity, 500 µM of Acetyl-CoA and 500 µM of GlcN-6P were used in a solution of 25 mM Tris-HCl–150 mM NaCl, pH 7.2 and 1 or 10 µg/mLof Apicomplexa GNA1 proteins were added to start the reaction in 50 µl. Reactions were stopped after 30 min with 50 µl of stop solution (25 mM bis-Tris-propane, 250 mM NaCl, 2 mM EDTA, and 6.4 M guanidine chloride, pH 7.5). Another 50 µl of DTNB solution (1 mM DTNB in 0.1% dimethyl sulfoxide, 25 mM Tris-HCl, 250 mM NaCl, and 2 mM EDTA, pH 7.5) was added to the reaction. The reaction of DTNB with CoASH (by-product of GlcN-6P acetylation) generates 2-nitro-5-thiobenzoate anion, whose absorbance can be read at 412 nm (Epoch Microplate Spectrophotometer, BioTek). To calculate kinetic constants, different concentrations up to 3 mM of AcCoA or GlcN-6P were used for *Cp*GNA1. Data were analyzed with GraphPad Prism 7. To detect SSAT activity the same protocol was used using as substrate 500 µM of Spermine or Spermidine and 3 different concentrations of *Cp*GNA1 (1, 20 and 500 µg/mL).

References

1. Edgar RC. MUSCLE: multiple sequence alignment with high accuracy and high throughput. Nucleic Acids Res. 2004;32:1792–7

2. Jones DT, Taylor WR, Thornton JM. The rapid generation of mutation data matrices from protein sequences. Comput. Appl. Biosci. 1992;8:275–82

3. Darriba D, Taboada GL, Doallo R, Posada D. ProtTest 3: fast selection of best-fit models of protein evolution. Bioinformatics 2011;27:1164–5

4. Ronquist F, Huelsenbeck JP. MrBayes 3: Bayesian phylogenetic inference under mixed models. Bioinformatics 2003;19:1572–4

5. Guindon S, Dufayard J-F, Lefort V, Anisimova M, Hordijk W, Gascuel O. New algorithms and methods to estimate maximum-likelihood phylogenies: assessing the performance of PhyML 3.0. Syst. Biol.. 2010;59:307–21

6. Anisimova M, Gascuel O. Approximate likelihood-ratio test for branches: A fast, accurate, and powerful alternative. Syst. Biol. 2006;55:539–52

7. Gouy M, Guindon S, Gascuel O. SeaView version 4: A multiplatform graphical user interface for sequence alignment and phylogenetic tree building. Mol. Biol. Evol. 2010;27:221–4

8. Letunic I, Bork P. Interactive tree of life (iTOL) v3: an online tool for the display and annotation of phylogenetic and other trees. Nucleic Acids Res. 2016;44:W242-5

9. Bailey TL, Boden M, Buske FA, Frith M, Grant CE, Clementi L, et al. MEME SUITE: tools for motif discovery and searching. Nucleic Acids Res. 2009;37:W202-8

10. Castresana J. Selection of conserved blocks from multiple alignments for their use in phylogenetic analysis. Mol. Biol. Evol. 2000;17:540–52

11. Adamczak R, Porollo A, Meller J. SABLE protein structure prediction server 2003

12. Banerjee S, Robbins PW, Samuelson J. Molecular characterization of nucleocytosolic O-GlcNAc transferases of Giardia lamblia and Cryptosporidium parvum. Glycobiology 2009;19:331–6

13. Mariño K, Güther MLS, Wernimont AK, Qiu W, Hui R, Ferguson MAJ. Characterization, localization, essentiality, and high-resolution crystal structure of glucosamine 6-phosphate N-acetyltransferase from Trypanosoma brucei. Eukaryot. Cell 2011;10:985–97

14. Hurtado-Guerrero R, Raimi OG, Min J, Zeng H, Vallius L, Shepherd S, et al. Structural and kinetic differences between human and *Aspergillus fumigatus* D-glucosamine-6-phosphate *N* -acetyltransferase. Biochem. J. 2008;415:217–23

SUPPLEMENTARY FIGURE LEGENDS

Fig. S1: High-resolution pairwise synteny analysis of the genomic regions containing the *GNA* gene in *Plasmodium falciparum strain 3D7* and 10 additional *Plasmodium* species using the CoGe tool GEvo. 2 Mb view. High-scoring segment pairs (HSPs) are shown by colored bars on top of the *P. falciparum*, which was used as reference genome in all pairwise comparisons after masking non-coding sequences. *GNA* genes are enclosed within a black circle. Note, in each comparison, the series of collinear genes between the two genomic regions, suggesting their common evolutionary origin. These analyses may be regenerated following the link https://genomevolution.org/r/snt9.

Fig. S2: High-resolution pairwise synteny analysis of the genomic regions containing the *GNA* gene in *Plasmodium falciparum strain 3D7* and four additional Apicomplexa species using the CoGe tool GEvo. 2 Mb view. High-scoring segment pairs (HSPs) are shown by colored bars on top of the *P. falciparum*, which was used as reference genome in all pairwise comparisons after masking non-coding sequences. *GNA* genes are enclosed within a black circle. Note, in each comparison, the series of collinear genes between the two genomic regions, suggesting their common evolutionary origin. These analyses may be regenerated following the link https://genomevolution.org/r/snst.

Fig. S3. Multiple sequence alignment of the conserved regions of the GNAT domain in apicomplexan and non-apicomplexan eukaryote GNA1s. For the complete list of sequences examined in this alignment see Supplementary Tables S5 and S7. The consensus sequence with the positions conserved in at least 60, 75 or 90 % of pairwise comparisons of the three protein families is shown below. The columns are colored according to amino acid physico chemical properties. The location of the consensus motif for GNC5-related *N*-acetyltransferase family is underlined.

Fig. S4. Secondary structure of six Apicomplexa GNA1. The protein secondary structures were predicted using SABLE. The locations of the GNAT domain and the consensus motif [(R/Q)-X-X-Q-X-G] of GNC5-related *N*-acetyltransferase protein sequences are indicated with single and double underline, respectively.

Fig. S5. Residual enzymatic activity of *P. falciparum* and *T. annulata* GNA1. Purified recombinant *Pf*GNA1 (left) and *Ta*GNA1 (right) enzymes, unstable and with a low level of soluble expression, were assayed in the presence of GlcN-6P and acetyl-CoA. GlcNAc-6P was quantified by multiple reaction monitoring LC-MS/MS.

Fig. S6. GNA enzymatic activity monitored by 2-nitro-5-thiobenzoate generation. *Cp*GNA1, *Et*GNA1, *Gn*GNA1, *Bb*GNA1 and *Tg*GNA1 are able to acetylate glucosamine-6-phosphate (GlcN-6P) generating *N*-acetylglucosamine-6-phosphate (GlcNAc-6P) *in vitro*. All reactions were allowed to proceed for 45 minutes with 50 and 500 ng of recombinant GNA1. The enzymatic assay was performed in triplicate, absorbance at 412 nm was determined and the results were averaged.

Fig. S7. *C. parvum* GNA kinetics and colorimetric SAT/SSAT assays. (A) *Cp*GNA1 kinetic assay plots are shown using different concentrations of acetyl-CoA and a fixed (500 µM) concentration of GlcN-6P (left) and with variable concentrations GlcN-6P and a fixed (500 µM) concentration of acetyl-CoA (right). (B) *Cp*GNA1 colorimetric assays using 500 µM GlcN-6P to detect GNA1 activity, or 500 µM spermine or 500 µM spermidine to detect SSAT activity. 50 ng, 1 µg or 25

µg of *Cp*GNA1 enzyme were used in a final volume of 50 µl. (C) *Cp*GNA1 and *Tg*GNA1 purified recombinant enzymes assayed with GlcN-6P and acetyl-CoA in the presence of 0.5 mM and 1.5 mM of spermine (open bars) and spermidine (grey bars), to determine putative spermine/spermidine inhibitory activity on GNA1 enzymatic assay. GlcNAc-6P was quantified by multiple reaction monitoring LC-MS/MS.

Figure S1

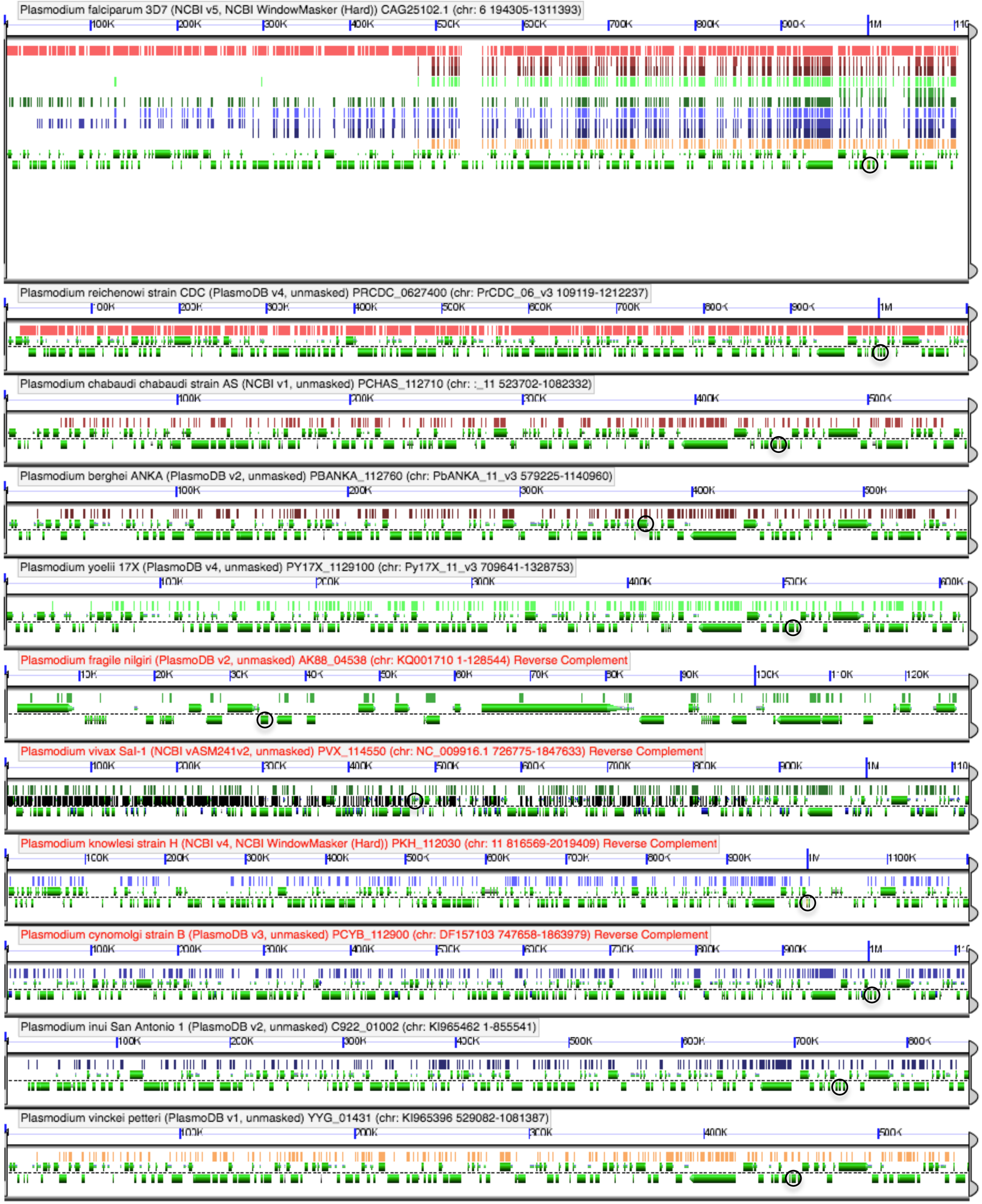

The Apicomplexa-specific glucosamine-6-phosphate N-acetyltransferase gene family encodes a key enzyme for glycoconjugate synthesis with potential as therapeutic target

M. Cova, B. López-Gutiérrez, S. Artigas-Jerónimo, A. González-Díaz, G. Bandini, S. Maere, L. Carretero-Paulet and L. Izquierdo

Figure S2

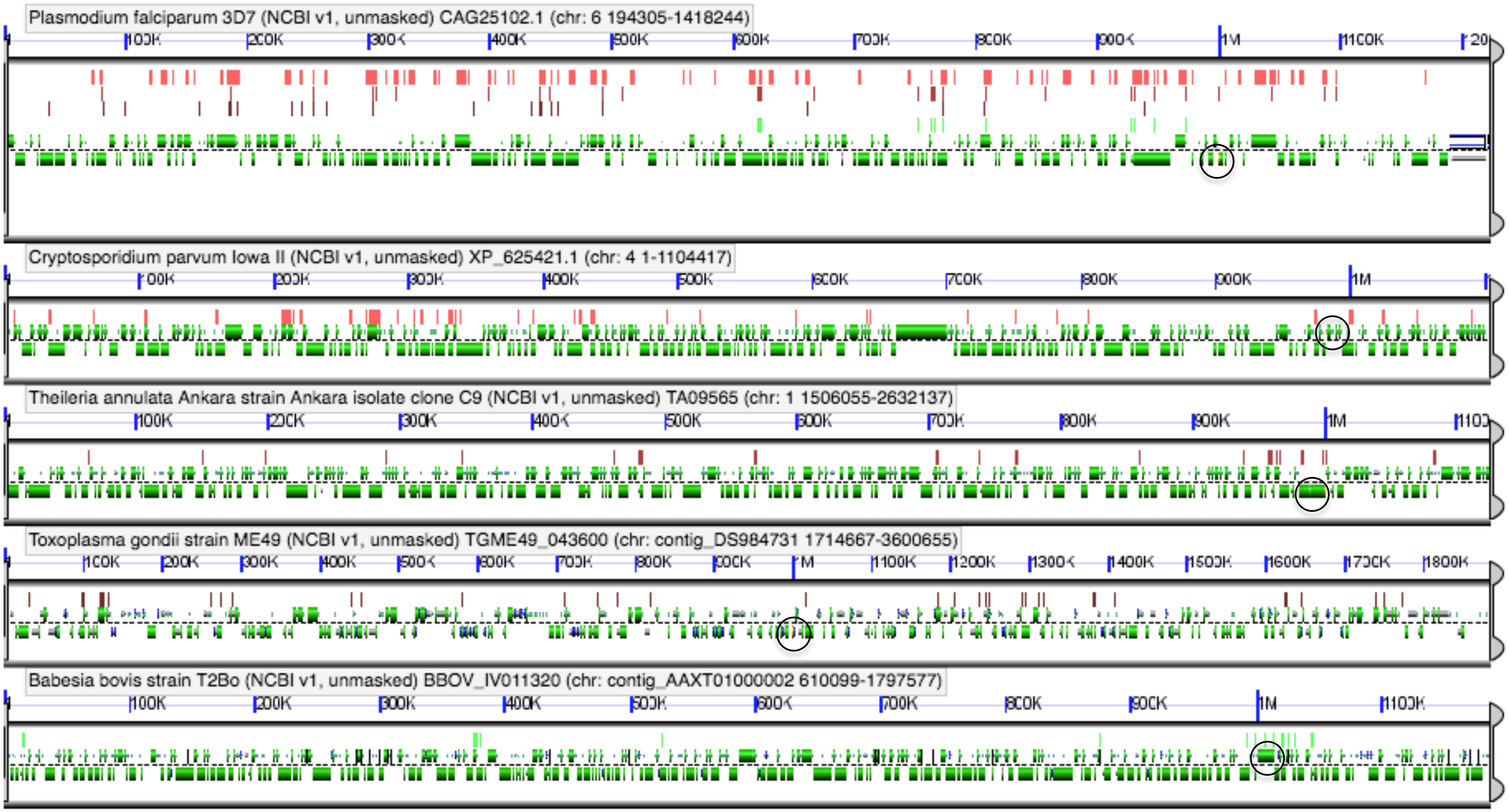

The Apicomplexa-specific glucosamine-6-phosphate N-acetyltransferase gene family encodes a key enzyme for glycoconjugate synthesis with potential as therapeutic target

M. Cova, B. López-Gutiérrez, S. Artigas-Jerónimo, A. González-Díaz, G. Bandini, S. Maere, L. Carretero-Paulet and L. Izquierdo

Figure S3

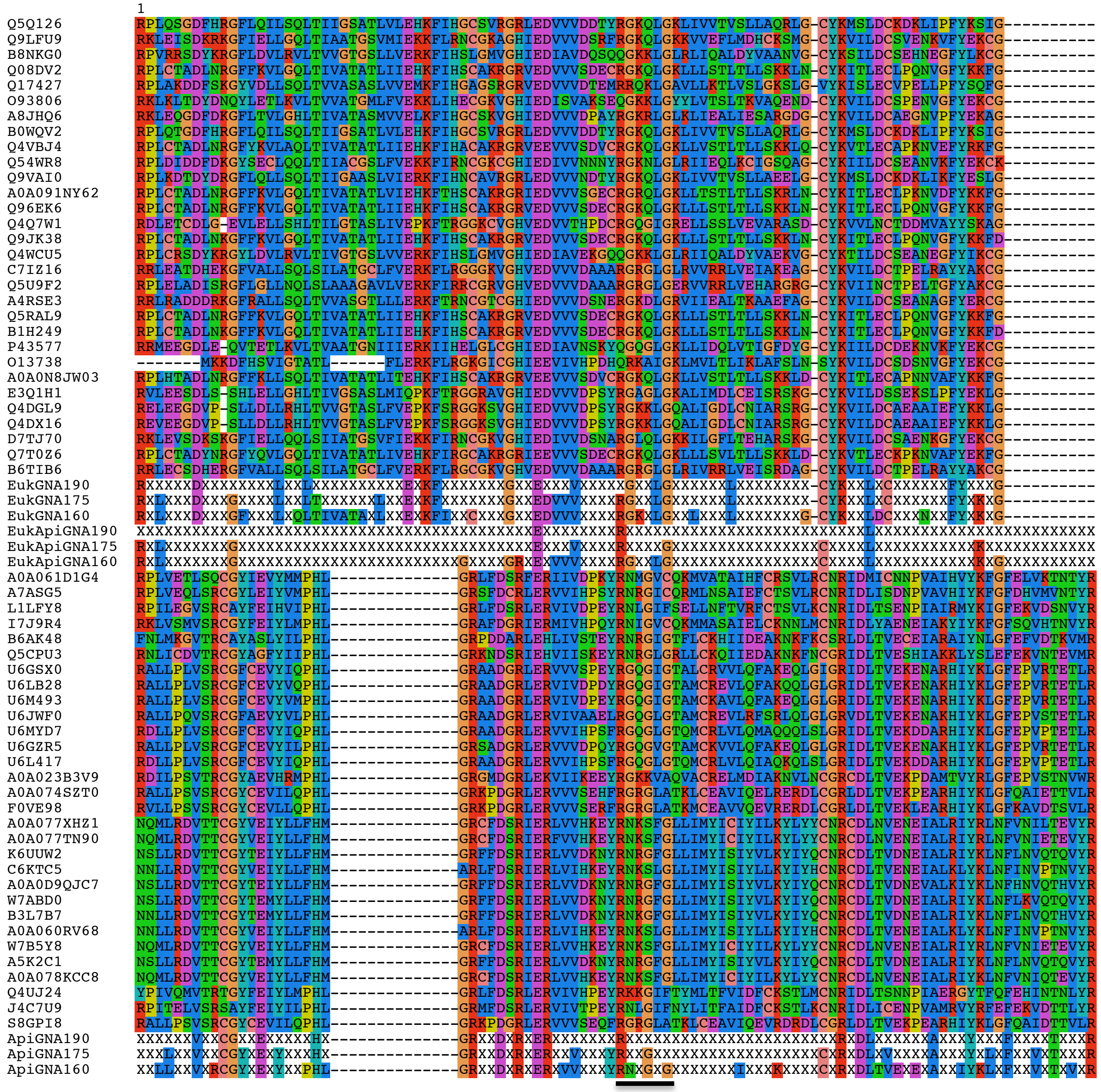

The Apicomplexa-specific glucosamine-6-phosphate N-acetyltransferase gene family encodes a key enzyme for glycoconjugate synthesis with potential as therapeutic target

M. Cova, B. López-Gutiérrez, S. Artigas-Jerónimo, A. González-Díaz, G. Bandini, S. Maere, L. Carretero-Paulet and L. Izquierdo

Figure S4

The Apicomplexa-specific glucosamine-6-phosphate N-acetyltransferase gene family encodes a key enzyme for glycoconjugate synthesis with potential as therapeutic target

M. Cova, B. López-Gutiérrez, S. Artigas-Jerónimo, A. González-Díaz, G. Bandini, S. Maere, L. Carretero-Paulet and L. Izquierdo

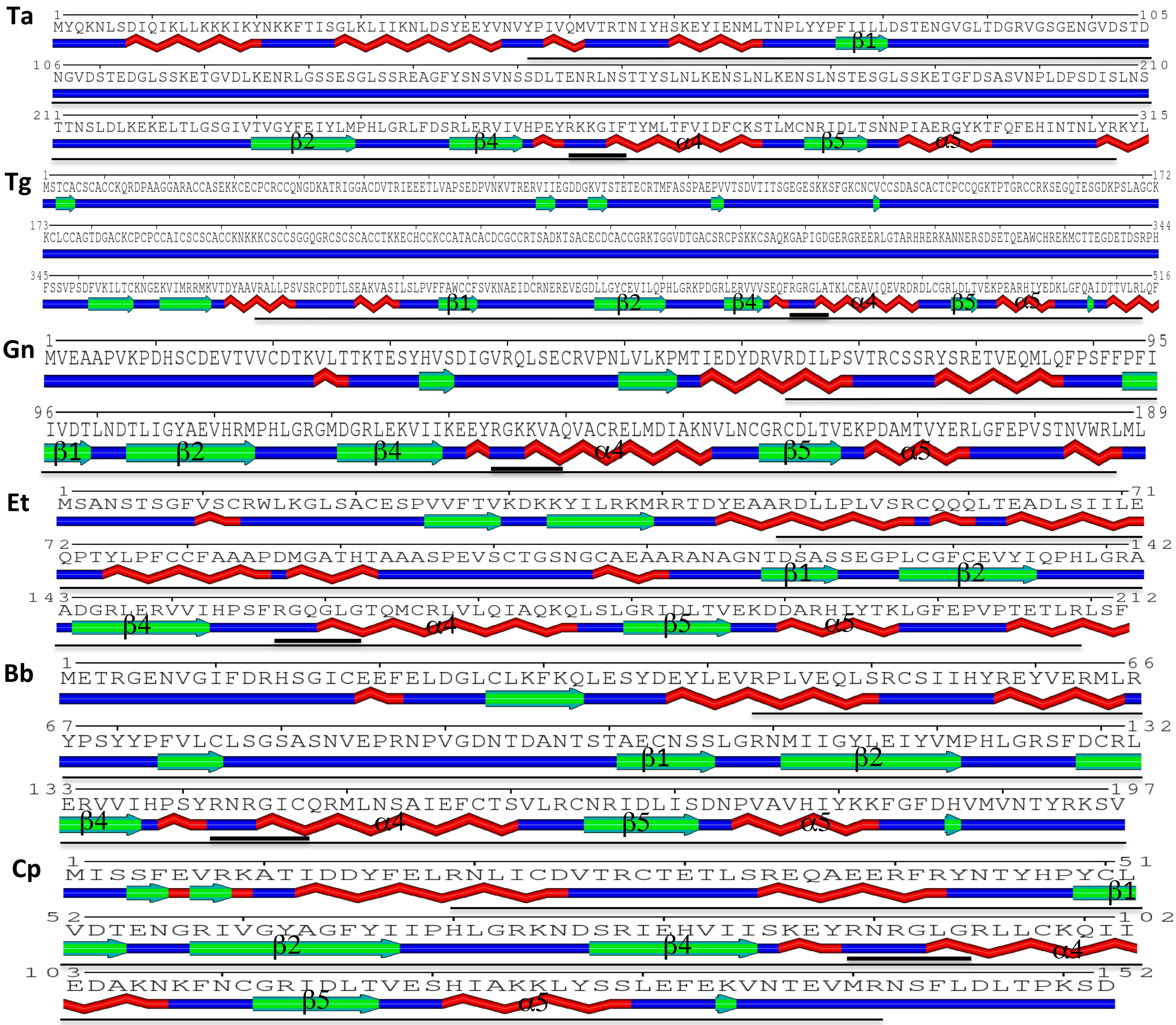

Figure S5

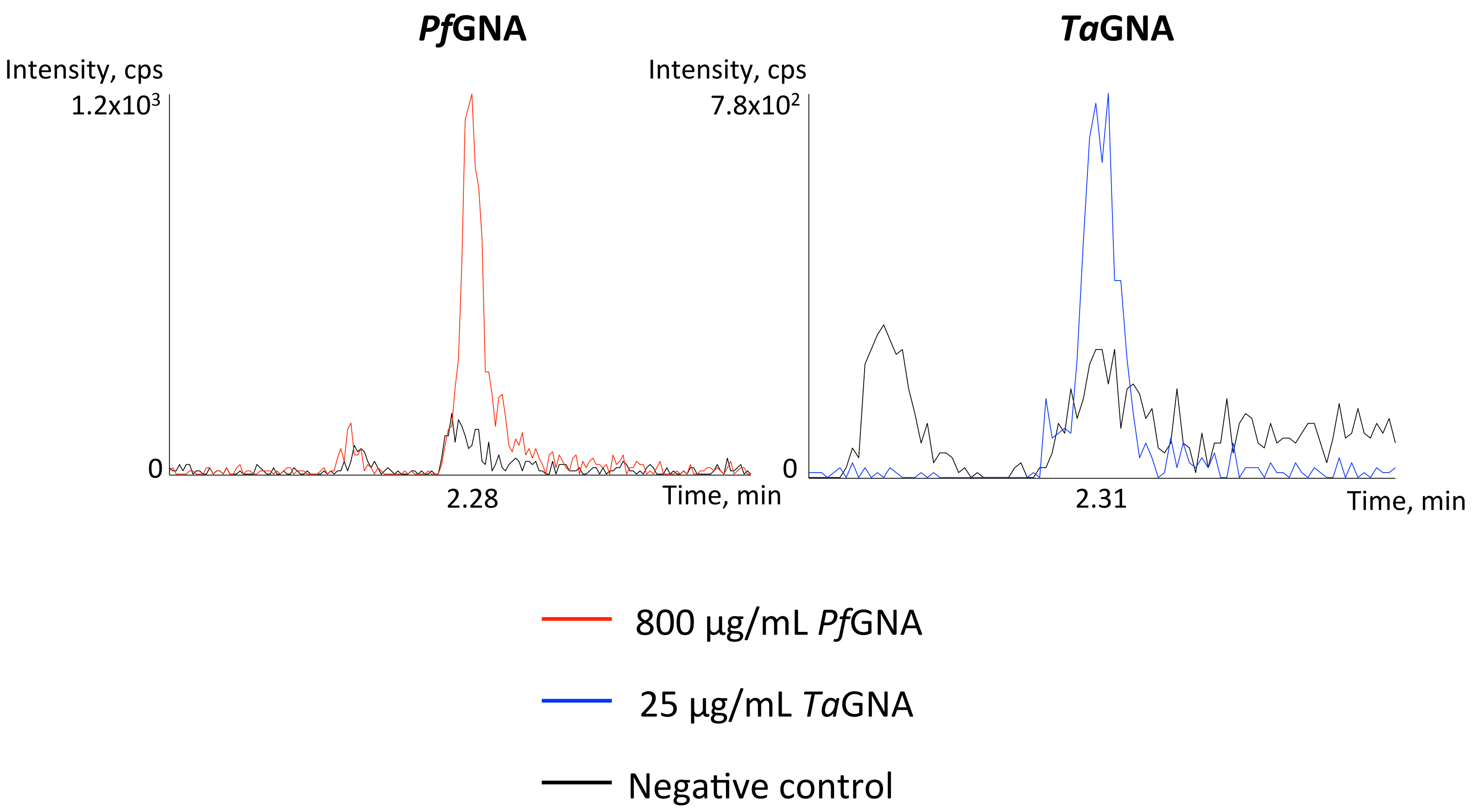

The Apicomplexa-specific glucosamine-6-phosphate N-acetyltransferase gene family encodes a key enzyme for glycoconjugate synthesis with potential as therapeutic target

M. Cova, B. López-Gutiérrez, S. Artigas-Jerónimo, A. González-Díaz, G. Bandini, S. Maere, L. Carretero-Paulet and L. Izquierdo

Figure S6

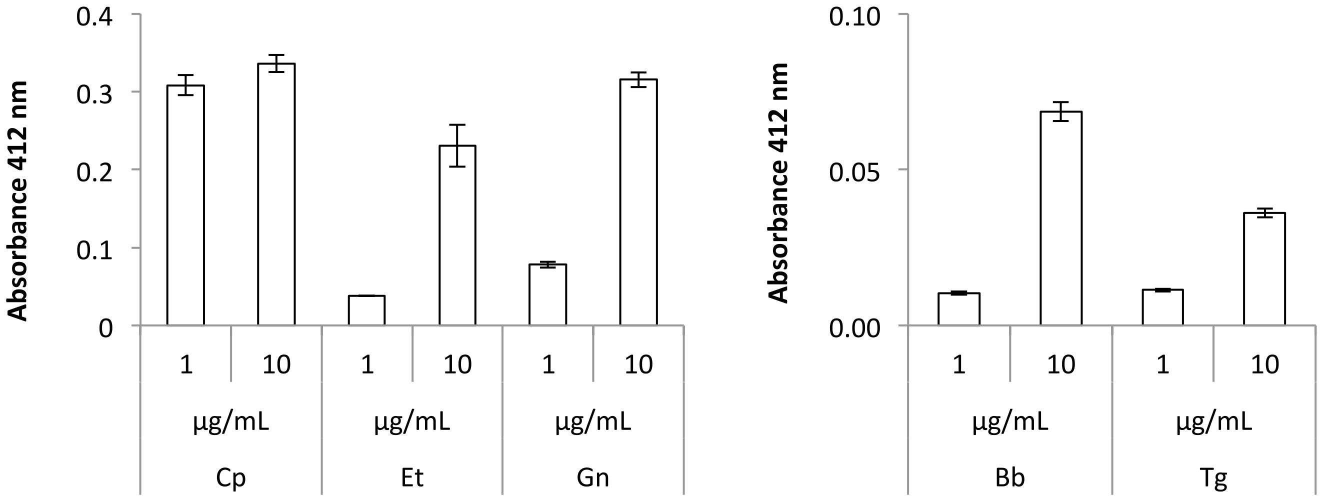

The Apicomplexa-specific glucosamine-6-phosphate N-acetyltransferase gene family encodes a key enzyme for glycoconjugate synthesis with potential as therapeutic target

M. Cova, B. López-Gutiérrez, S. Artigas-Jerónimo, A. González-Díaz, G. Bandini, S. Maere, L. Carretero-Paulet and L. Izquierdo

Figure S7

The Apicomplexa-specific glucosamine-6-phosphate N-acetyltransferase gene family encodes a key enzyme for glycoconjugate synthesis with potential as therapeutic target

M. Cova, B. López-Gutiérrez, S. Artigas-Jerónimo, A. González-Díaz, G. Bandini, S. Maere, L. Carretero-Paulet and L. Izquierdo

A

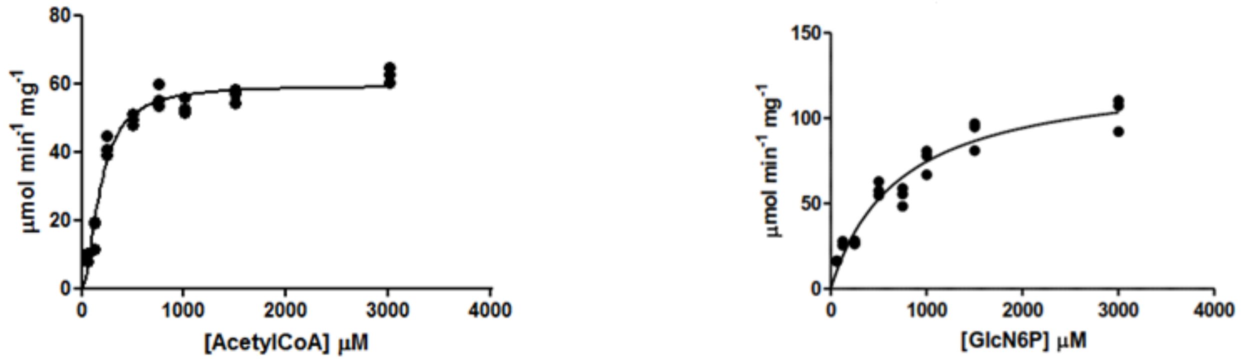

Parameter	Value	Std. Error
Vmax (μmol min^{-1} mg^{-1})	68.8	2.7
Km (μM)	241.7	35.3

Parameter	Value	Std. Error
Vmax (μmol min^{-1} mg^{-1})	128.1	8.4
Km (μM)	719.1	119.0

B

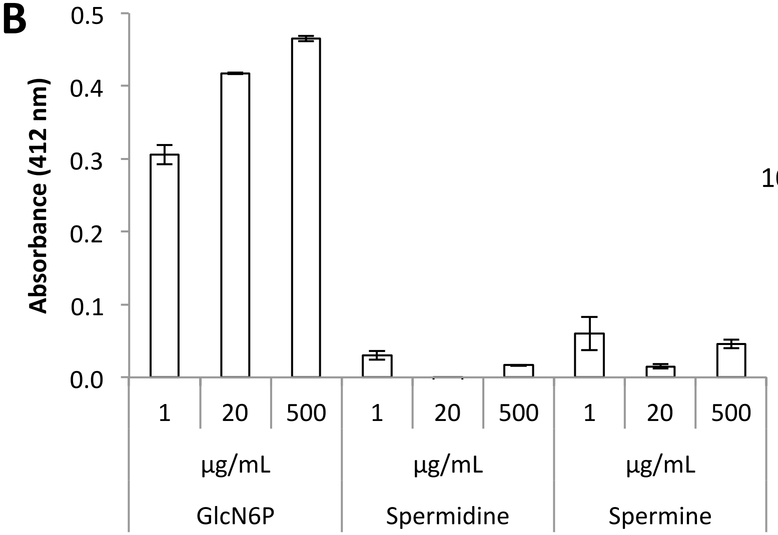

C

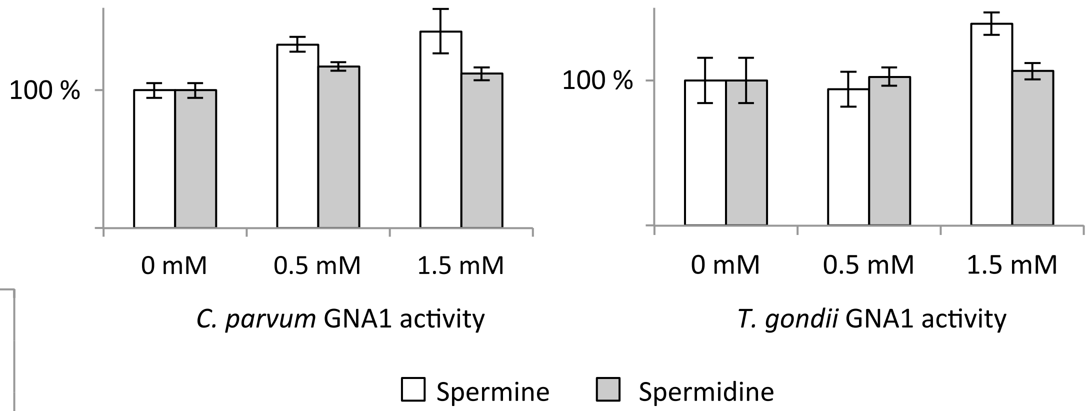

SUPPLEMENTARY TABLE LEGENDS

Table S1. Summary of 71 GNAT-domain containing proteins from seven apicomplexan organisms identified in this study and their orthogroup classification according to the EupathDB database.

Table S2. Summary of 18 orthogroups plus one singleton, as defined by EupathDB databases, clustering 71 GNAT-domain containing proteins from seven apicomplexan organisms identified in this study, and their taxonomic distribution. Orthogroups retrieved as well-supported clades in our phylogenetic analysis are highlighted in bold.

Table S3. Summary of 20 conserved motifs identified by MEME across 71 GNAT-domain containing protein sequences from seven apicomplexan organisms. The settings used for MEME's detection of motifs are shown in the first row. Correspondence of the best possible match sequence with INTERPRO protein functional domains is shown for each motif.

Table S4. Enzymes involved in sugar nucleotide pathways, GPI-anchor and *C*-, *N*- and *O*-glycan biosynthesis in *P. falciparum*, and their taxonomic distribution. For each protein, the taxonomic distribution of the corresponding orthogroup as defined in the OrthoDB database is shown for 12 different taxonomic groups grouping a total of 150 species. Numbers indicate the fraction of species in each taxonomic group with at least one representative in Fir, Firmicutes (out of 6 species); Pro, Proteobacteria (19); Oba, Other bacteria (11); Arc, Archaea (16); Eug, Euglenozoa (9); Amo, Amoebozoa (4); Vir, Viridiplantae (11); Tet, *Tetrahymena thermophila* (1); Api, Apicomplexa (14); Fun, Fungi (24); Met, Metazoa (29); Oeu, Other eukaryotes (6). The bottom row indicates the total number of species within each taxonomic group. The corresponding co-orthologs in human and six additional species from Apicomplexa, as defined by orthogroup membership and best reciprocal hit (BRH), is shown. BRH were defined as the best hit resulting from BLASTP and DELTA-BLAST (Domain Enhanced Lookup Time Accelerated BLAST) searches in the corresponding proteome using the *P. falciparum* protein sequence as query with an E-value cutoff of 1E-5 (*), when the original *P. falciparum* query was retrieved as first hit in reciprocal searches against the respective human or Apicomplexa genome. The co-orthologs retrieved by BRH corresponded in all cases to the one retrieved in the orthogroup, except for Phosphatidylinositol *N*-acetylglucosaminyltransferase subunit H, for which the corresponding orthogroup did not include any animal representative. Please, note that this table is not meant to provide with comprehensive list of all enzymes involved in sugar nucleotide and related biosynthetic pathways in the Apicomplexa phylum, as occurrence of lineage-specific gene acquisition or losses cannot be excluded.

Table S5. Summary of GNA1 proteins from 30 apicomplexan species identified in this study. Both EupathDB gene names and UNIPROT entry names are shown.

Table S6. Summary of 11 conserved motifs identified by MEME across 30 GNA1 protein sequences from Apicomplexa. The settings used for MEME's detection of motifs are shown in the first row. Correspondence of the best possible match sequence with INTERPRO functional domains is shown for each motif.

Table S7. Summary of 30 GNA1 sequences from non-apicomplexan eukaryote organisms.

Table S8. List of primers used in this study.
